# Nutritional Care After Hospital Discharge (NutriCAD): protocol for a stepped-wedge, multicentre, randomised controlled trial in paediatrics

**DOI:** 10.1136/bmjopen-2026-122183

**Published:** 2026-10-27

**Authors:** Esther van Steenbergen, Doortje Hage, Ankie Lebon, Herbert van Wering, Janneke Stapelbroek, Eefje Winder-Bakker, Renske Bottema, Merel Baart-Visser, Sandra Van Gils, Evi Van den Berg, Esmée Kroon, Yolah Soldaat, Marten J Poley, Sten Willemsen, Jessie Hulst, Angelika Kindermann, K Dulfer, Elles van der Louw, K Joosten, Sascha Verbruggen

**Affiliations:** 1 Department of Neonatal and Paediatric Intensive Care, Erasmus MC, Rotterdam, Netherlands; 2 Internal Medicine, Division of Dietetics, Erasmus MC, Rotterdam, Netherlands; 3 Department of Paediatrics, Albert Schweitzer Hospital, Dordrecht, Netherlands; 4 Department of Paediatrics, Amphia Ziekenhuis, Breda, North Brabant, Netherlands; 5 Department of Paediatrics, Catharina Hospital, Eindhoven, Netherlands; 6 Department of Dietetics, Noordwest Ziekenhuisgroep, Alkmaar, Netherlands; 7 Department of Paediatrics, Gelderse Vallei Hospital, Ede, Netherlands; 8 Department of Dietetics, Albert Schweitzer Hospital, Dordrecht, Netherlands; 9 Department of Dietetics, Amphia Hospital, Breda, Netherlands; 10 Department of Dietetics, Catharina Hospital, Eindhoven, Netherlands; 11 Department of Dietetics, Gelderse Vallei Hospital, Ede, Netherlands; 12 Institute for Medical Technology Assessment (iMTA), Erasmus University Rotterdam, Rotterdam, Netherlands; 13 Obstetrics and Gynaecology, Erasmus MC, Rotterdam, Netherlands; 14 Biostatistics, Erasmus MC, Rotterdam, Netherlands; 15 Department of Paediatrics, Division of Gastroenterology, Hepatology and Nutrition, SickKids, Toronto, Ontario, Canada; 16 Department of Paediatrics and Department of Nutritional Sciences, University of Toronto, Toronto, Ontario, Canada; 17 Department of Paediatrics, Division of Gastroenterology, Hepatology, Amsterdam UMC Locatie AMC, Amsterdam, Netherlands; 18 Department of Child and Adolescent Psychiatry/Psychology, Erasmus MC, Rotterdam, Netherlands; 19 Department of Internal Medicine, Erasmus MC, Rotterdam, Netherlands

**Keywords:** Paediatric intensive & critical care, Patient Care Management, Randomized Controlled Trial, NUTRITION & DIETETICS, Paediatric gastroenterology

## Abstract

**Introduction:**

In the Netherlands, an estimated 60 000–70 000 children are hospitalised annually, of whom approximately 9% are acutely malnourished or at high risk at the time of admission. Every year, 3000 children are discharged with ongoing nutritional support. Disjointed postdischarge care often leads to prolonged enteral feeding dependency, feeding difficulties and nutritional deterioration. A structured, multidisciplinary follow-up programme may improve nutritional outcomes, reduce healthcare utilisation and support family well-being. The Nutritional Care After Hospital Discharge (NutriCAD) study aims to evaluate whether a tailored, multidisciplinary nutritional care programme with a parent empowerment component is cost-effective and improves growth, feeding behaviour and parental well-being in children discharged from hospital with nutritional support.

**Methods and analysis:**

NutriCAD is a stepped-wedge cluster randomised controlled trial conducted in one academic and five general hospitals in the Netherlands. 260 children (term-born neonates to 18 years) discharged with newly initiated enteral nutritional support will be enrolled. At baseline, all hospitals deliver usual care; clusters sequentially cross over to the intervention at 3 monthly intervals. The intervention comprises an individually tailored nutritional plan and a structured parent empowerment programme, delivered at discharge and at 6-week, 12-week and 18-week outpatient follow-up visits. The primary outcomes are change in weight-for-age SD-score (children <1 year) or weight-for-height SD-score (children ≥1 year) and change in height-for-age SD-score from discharge to 6 months postdischarge. Secondary outcomes are changes in body composition, duration and frequency of nutritional support and/or dependency on nasogastric tube feeding, nutritional requirements and intake, feeding behaviour of the child, parental stress, quality of life of children and parents, cost-effectiveness of tailored nutritional care and barriers and facilitators of implementation of the multidisciplinary team 6 months after discharge. Analyses will follow the intention-to-treat principle using mixed-effects regression models, adjusted for clustering and predefined confounders.

**Ethics and dissemination:**

The protocol and informed consent forms were approved by the Institutional Review Board of Erasmus MC, Rotterdam (MEC-2023-0490). Formal ethical approval was waived at all other participating sites, each of which obtained local Board of Directors authorisation. Written informed consent is obtained from parents or legal guardians; participants aged 12–15 years also provide written assent, and participants aged 16 years and older provide their own written consent. Results will be disseminated through peer-reviewed publications and national and international scientific conferences.

**Trial registration number:**

NCT06603792.

**STRENGTHS AND LIMITATIONS OF THIS STUDY**

- This pragmatic, multicentre stepped-wedge, cluster randomised controlled trial, implemented across one academic and five general hospitals, minimises contamination risk while ensuring all sites and patients ultimately receive the intervention.
- The study assesses a broad range of nutritional outcomes, extending beyond standard anthropometrics.
- It includes both child outcomes and parental quality of life and stress, providing a more complete view of the intervention’s impact.
- The stepped-wedge design restricts inclusion flexibility; if recruitment targets are not met in time, extending the inclusion period is not possible.

## Introduction

 Malnutrition remains a substantial concern in paediatric hospital care. In the Netherlands, 60 000–70 000 children are admitted annually, with approximately 9% acutely malnourished or at risk on admission.^1–4^ Malnutrition is associated with prolonged recovery, impaired growth and neurodevelopment, and reduced quality of life (health-related quality of life (HRQoL)) for children and families.^5–11^ The condition contributes to significant healthcare and societal costs.^12^

Artificial nutritional support such as oral supplements or enteral tube feeding is commonly initiated during hospitalisation.^13^ In the Netherlands, around 3000 children each year leave the hospital with ongoing nutritional support.^14^ Our recent multicentre study in tertiary paediatric care demonstrated that 35.9% of children receiving in-hospital nutritional support were discharged with continuing nutritional care. Critically, more than 70% were still receiving nutritional support 6 months after discharge.^15^

Despite this high prevalence, follow-up pathways are often fragmented; care is distributed across multiple clinicians and settings, leading to inconsistent management and sometimes unnecessarily prolonged nutritional support.^5 15^

Extended reliance on tube feeding or supplementation increases the risk of feeding disorders,^16^ as children may miss critical developmental experiences essential for oral skill acquisition. Recovery of normal eating patterns may take up to 2 years.^17^

Parents report feeding problems after discharge as a primary stressor, surpassing distress from the underlying medical condition itself.^18 19^ Moreover, adjusted nutritional regimens may also accelerate weight gain, increasing long-term metabolic consequences.^20 21^

These findings highlight the need for targeted nutritional support after discharge.

A structured, multidisciplinary follow-up programme has the potential to improve nutritional intake, shorten the time to full oral feeding, optimise nutritional and growth trajectories and reduce parental stress. Despite requiring investment in specialist dietitian, psychologist and paediatrician time, such a programme could be cost-effective by reducing downstream healthcare consumption.

We hypothesise that in children discharged from hospital with ongoing nutritional support, a tailored nutritional plan combined with structured multidisciplinary follow-up and parent empowerment programme, delivered at discharge and at 6-week, 12-week and 18-week follow-up, will: (1) be cost-effective; (2) improve nutritional intake, nutritional status and feeding behaviour of the child; and (3) improve emotional functioning and reduce parental stress, 6 months after discharge. The primary aim of the Nutritional Care After Hospital Discharge (NutriCAD) study is to investigate whether a tailored, multidisciplinary nutritional care follow-up programme for children discharged from hospital with nutritional support improves nutritional intake and status, feeding behaviour and quality of life in both children and their parents, compared with usual care.

## Methods and analysis

### Study design

The design was guided by findings from a pilot study conducted at Erasmus MC Sophia Children’s Hospital,^15^ which highlighted high rates of ongoing post nutritional care. The NutriCAD study is a prospective, stepped-wedge cluster randomised controlled trial (SW-CRCT) conducted in one academic hospital (Erasmus MC Sophia Children’s Hospital, Rotterdam) and five general hospitals across the Netherlands (Albert Schweitzer Hospital, Dordrecht; Amphia, Breda; Catharina Hospital, Eindhoven; Noordwest Hospital Group, Alkmaar; and Hospital Gelderse Vallei, Ede). A comparison of participating hospitals with respect to annual paediatric admissions, level of care and site characteristics is provided in online supplemental table 1. Design and reporting follow Consolidated Standards of Reporting Trials (CONSORT) guidelines for stepped-wedge trials.^22^ In this design, all hospitals begin in the control phase and sequentially cross over to the intervention at 3-month intervals, ensuring each cluster experiences both conditions (figure 1). The design was selected in preference to a parallel group CRCT, because it minimises contamination risk and allows all sites eventual access to the intervention, which is considered ethically justifiable given its anticipated benefit. Each step of 3 months incorporates a 1-month implementation period to allow the intervention to be integrated into routine care.

**Figure 1:**
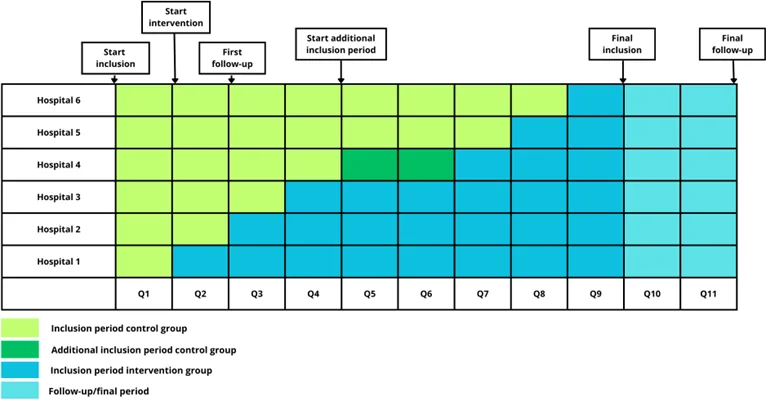
Stepped-wedge study design and timeline, including follow-up.

### Randomisation and allocation

Randomisation operates at the cluster level according to the stepped-wedge schedule; participants thus receive whichever condition (control or intervention) is active at their hospital during enrolment. Blinding of families and clinicians is not feasible, but outcome assessors performing data cleaning and statistical analysis remain blinded where possible.

### Participant eligibility criteria

Children from term-born neonates (gestational age ≥37 weeks) up to 18 years, who: (1) were admitted to a participating hospital and had nutritional support (oral nutritional supplements and/or enteral tube feeding) newly initiated during that admission; and (2) are discharged home with continuation of nutritional support.

Children will be excluded if they: (1) were already receiving nutritional support at the time of hospital admission (ie, pre-existing nutritional support); (2) require parenteral nutrition at the time of discharge; (3) carry a Diagnostic and Statistical Manual of Mental Disorders, Fifth Edition diagnosis, eating or feeding disorder (eg, avoidant/restrictive food intake disorder, anorexia nervosa) prior to this admission; or (4) have a parent/legal guardian who is unable to provide written informed consent.

### Recruitment and informed consent

Eligible children will be identified during admission by the paediatric care team. The study will be explained to parents by a trained member of the research team, and when appropriate to the child. Written informed consent will be obtained before discharge. Certified interpreters will be used in case of language barriers. Consent and assent procedures are differentiated by age per Dutch law (Medical Research Involving Human Subjects Act (WMO)): participants under 12 years—written consent from parents/legal guardians; 12–15 years—parental consent plus written assent from the participant; 16 years and older—written consent from the participant themselves. For participants aged 16 and older, the parent empowerment programme is adapted to focus on participant nutritional self-management.

### Calculation of sample size

The sample size calculation is based on the co-primary endpoints of change in weight-for-age SD-score (WFA-SDS, for children<1 year) and weight-for-height SD-score (WFH-SDS, for children ≥1 year). These coprimary endpoints represent age-stratified measures of the same construct (nutritional status); the power calculation was therefore based on a single unified effect size applied across both age strata. No correction for multiple comparisons was applied, consistent with established practice for coprimary endpoints measuring the same construct. In a recent pilot study of 206 children conducted at Erasmus MC Sophia Children’s Hospital (the trial’s academic centre), children receiving postdischarge nutritional support showed a mean weight-for-age improvement of 0.4 SD (from −0.7 SD at admission to −0.3 SD at 4–6 months follow-up).^15^ This cohort is regarded as representative of the usual care control population in the current trial.

On this basis, the sample size required to detect a clinically meaningful additional improvement of 0.8 SD in the intervention group compared with the control group (ie, intervention group improvement ≈1.2 SD vs control group ≈0.4 SD), with 80% statistical power at a two-sided α=0.05, is n=196 patients.

To account for the clustered nature of the data in the SW-CRCT, a variance inflation factor (VIF) was applied using the formula VIF=1 + ρ(m − 1), where ρ is the intracluster correlation coefficient and m is the mean cluster size. The intracluster correlation was set at ρ=0.01, consistent with published estimates for hospital-based paediatric interventions and mean cluster size m=196/6 ≈ 33. This yields VIF=1 + 0.01 × 32 = 1.32. The design-adjusted sample size is therefore 1.32×196 ≈ 260 patients.

Following a protocol amendment after 9 months of enrolment, necessitated by a slower-than-anticipated recruitment rate, two additional inclusion periods were inserted into the middle of the original stepped-wedge schedule (figure 1). Extension of the inclusion period at the end of the trial was not feasible, as this would have resulted in an imbalance between intervention and control observations, reducing statistical efficiency. A simulation study based on the observed recruitment rate estimated that, without this amendment, effective statistical power would be 39%; with the inserted periods, power is estimated at 53%. The amendment was deemed the most appropriate and feasible design adaptation within the logistical and financial constraints of the study. The original stepped-wedge schedule was designed based on the maximum feasible sample size within the available funding period and projected patient volume across participating hospitals. The a priori power of 39% was a consequence of these constraints rather than a predefined target; the trial was conceived as a necessary first empirical step in this understudied area, intended to inform a future, adequately powered confirmatory trial.

### Intervention

Participants allocated to the intervention condition receive a tailored, multidisciplinary nutritional care programme consisting of two complementary components: (1) an individually tailored nutritional plan; and (2) a structured parent empowerment programme.

#### Tailored nutritional plan

At discharge, the multidisciplinary team (dietitian, paediatrician, psychologist when indicated) cocreates an individualised nutritional plan specifying caloric and protein needs, micronutrient requirements, feeding schedules, type of formula or oral nutrition and criteria for weaning. The plan is documented in the medical record and shared with community healthcare providers.

#### Parent empowerment programme

Parents receive structured education on their child’s nutritional needs, tube feeding or oral nutritional supplements if applicable, recognition of feeding difficulties, safe weaning of nutritional support and when to contact healthcare professionals. Educational content is delivered in accessible language with written and digital support. Sessions are delivered by the paediatric dietitian at discharge and at each follow-up visit at 6, 12 and 18 weeks. For participants aged 16 and older, content focuses on participant self-management.

### Follow-up visits

Structured outpatient visits occur at 6, 12 and 18 weeks after discharge, including assessment of growth (anthropometrics), nutritional requirements versus intake via a food diary, assessing feeding difficulties and feeding behaviour of the child (questionnaires), and parental stress (questionnaire) (figure 2). Findings are integrated in a digital dashboard, which allows visualisation of key outcome trends. The nutritional plan is adjusted at each visit in response to the child’s clinical progress and the needs of the family. Visits may involve dietitians, paediatricians, psychologists, speech or occupational therapists, depending on the feeding problem’s underlying cause.

**Figure 2:**
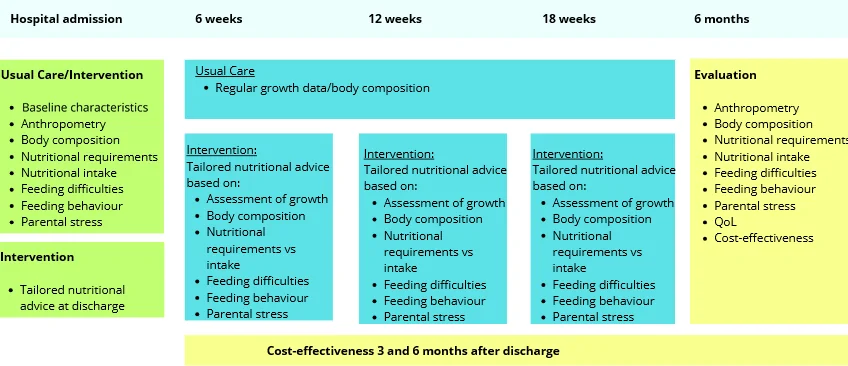
Study parameters from admission to 6 months after discharge. QoL, quality of life.

### Usual care (control condition)

During the control period, children receive standard nutritional care at discharge and during follow-up, based on each hospital’s usual practice. Usual care may differ between sites and reflects current paediatric practice in the Netherlands. In general, this consists of a written discharge letter with nutritional instructions to the general practitioner and/or community dietitian, without a structured multidisciplinary outpatient follow-up programme. At the academic centre (Erasmus MC Sophia Children’s Hospital), community dietitians receive a more detailed transfer document; at general hospital sites, discharge instructions vary by ward and clinician.

### Criteria for modifying or discontinuing the intervention

Participants may withdraw from the study at any time without affecting their clinical care. Clinicians may also withdraw participants for urgent medical reasons. No protocol-level stopping rules apply at the individual level, as the intervention is a structured nutritional care programme rather than a pharmacological treatment. Participants who discontinue the intervention will continue to receive usual care, and (if consent remains) primary outcome data will still be collected at 6 months postdischarge in line with the intention-to-treat principle.

### Adherence monitoring

Adherence is assessed by recording attendance at outpatient visits at 6, 12 and 18 weeks postdischarge. Completion of 3-day food diaries and validated questionnaires is tracked electronically via Erasmus MC Information and Communications Technology systems. For control participants, completion of the structured logbook is similarly monitored. Missed appointments will be followed up by the research team, and telehealth alternatives will be arranged where possible to maintain engagement and limit missing data.

### Concomitant care

No restrictions are placed on additional medical or nutritional care during the trial. Participants continue to receive care from their general practitioner, community dietitian and other providers as needed. Use of any new oral nutritional supplements or enteral formulas started after baseline will be recorded at each follow-up and included as covariates in sensitivity analyses where appropriate.

### Primary endpoints

Coprimary endpoints are: (1) change in WFA-SDS in children <1 year and (2) change in WFH-SDS in children ≥1 year, from discharge to 6 months postdischarge. Change in height-for-age SD-score over the same period is included to assess linear growth independently of weight gain.

### Anthropometric measurements

Standard measurements (weight, recumbent length (<2 years), standing height (≥2 years), head circumference (up to 24 months) and mid-upper arm circumference) will be obtained at each outpatient visit following published reference procedures.^23 24^ Trained personnel will perform all measurements according to site-specific standard operating procedures to ensure reliability and data quality. Anthropometric indices will be expressed as age-adjusted and sex-adjusted SD-scores (z-scores) using WHO Child Growth Standards,^25^ as summarised in table 1.

**Table 1:** Anthropometric indices used by age group

Age group	Anthropometric Index	Abbreviation
<1 year	Weight-for-age	WFA
<1 year	Length-for-age	LFA
<1 year	Head circumference-for-age	HCFA
≥1 year	Weight-for-height	WFH
≥1 year	Height-for-age	HFA
≥1 year	Body mass index-for-age	BMI-for-age

### Secondary outcome measures

Table 2 summarises all study outcomes, including measurement instruments, respondents and assessment time points across the follow-up period. Outcomes include anthropometry and body composition, nutritional intake and support characteristics, child feeding behaviour, parental psychological outcomes, child HRQoL and healthcare and societal costs. All outcomes were assessed at outpatient visits using validated instruments and standardised procedures (table 2). Further detailed descriptions of secondary outcome measures are reported in online supplemental material A.

**Table 2:** Summary of outcome measures, instruments and assessment time points

Domain	Instrument/measure	Respondent	Time points
Anthropometry	Weight, height/length, MUAC, HC	Clinician	Discharge; 6, 12, 18 wk; 6 mo
Body composition	Skinfolds^33 34^; InBody^35^; Pea Pod (age <2 years)/Bod Pod (age ≥2 years) (academic site)^36 37^	Clinician	Discharge; 6, 12, 18 wk; 6 mo
Nutritional requirements	Dutch Health council reference values; Energy,^38^ Protein,^39^ Vitamins; minerals^40^ and Screening and Treatment of Malnutrition in Children^41^	Clinician	Discharge; 6, 12, 18 wk; 6 mo
Nutritional intake	3-day food diary (Evry (2023))	Parent/caregiver/child (>16 years old)	Before each visit
Feeding difficulties	MCH-FS^42^	Parent/caregiver	Discharge; 6, 12 and 18 wk; 6 mo
Feeding behaviour (<1 year)	BEBQ^43^	Parent/caregiver	Discharge; 6, 12, 18 wk; 6 mo
Feeding behaviour (≥1 year)	CEBQ^44^	Parent/caregiver	Discharge; 6, 12, 18 wk; 6 mo
Parental stress	DT-P^45^	Parent/caregiver	Discharge; 6, 12, 18 wk; 6 mo
QoL: PTSD parent	PCL-5^46^	Parent/caregiver	6 mo
QoL: anxiety/depression parent	PROMIS Anxiety 8a; PROMIS Depression 8b^47^	Parent/caregiver	6 mo
QoL: child	GPROM^48^; CHU9D (age 5–17)^26^	Parent/child (>7 years old)	6 mo
Cost-effectiveness	CEA^49^; QALY^50 51^	Parent	18 wk
Cost-effectiveness: healthcare utilisation	iMCQ^52^	Parent	6, 12, 18 wk; 6 mo
Cost-effectiveness: productivity losses	iPCQ^53^	Parent	6, 12, 18 wk; 6 mo

BEBQ, Baby Eating Behaviour Questionnaire; CEA, cost-effectiveness analysis; CEBQ, Child Eating Behaviour Questionnaire; CHU9D, Child Health Utility 9D; DT-P, Distress Thermometer for Parents; GPROM, Generieke Patient Reported Outcome Measures; HC, head circumference; HRQoL, health-related quality of life; iMCQ, iMTA Medical Consumption Questionnaire; iPCQ, iMTA Productivity Cost Questionnaire; MCH-FS, Montreal Children’s Hospital Feeding Scale; mo, months; MUAC, mid-upper arm circumference; PCL-5, PTSD Checklist for Diagnostic and Statistical Manual of Mental Disorders, Fifth Edition; PROMIS, Patient-Reported Outcomes Measurement Information System; PTSD, post-traumatic stress disorder; QALY, quality-adjusted life year; QoL, quality of life; wk, weeks.

### Barriers and facilitators to multidisciplinary team implementation

To inform future implementation and scale-up, a nested qualitative substudy will be conducted alongside the trial. Semistructured, in-depth interviews (target n=8–12) will be conducted with healthcare professionals involved in the multidisciplinary nutrition support teams (lead clinicians, dietitians, psychologists and nurses). Interviews will be audio-recorded, transcribed verbatim and analysed using inductive thematic analysis to identify perceived barriers and facilitators to implementation and sustainability of the intervention.

### Other outcomes

#### Baseline characteristics

At baseline, demographic and clinical data will be collected, including age, sex, gestational age, birth weight, STRONGkids nutritional screening score, medication use, reason for admission, discharge diagnosis, underlying chronic conditions or syndromes and parental socio-economic status (education and occupation).

#### Characteristics during hospital stay

Nutritional support during admission will be recorded, including indication, type, timing and complications. Type of nutritional intake will be documented as breastmilk, formula or oral feeding. Macronutrient composition will be documented as: energy: kcal and % of energy requirements, protein: g/kg and/or EN%, fat: EN%, carbohydrate: EN% and major micronutrients if sufficient and description of deficient micronutrients.

Furthermore, at discharge the length of stay, admission to paediatric intensive care, duration of respiratory support, feeding-related medication started during hospital stay, anthropometric measurements, skinfold measurements and ‘InBody’ measurements of fat mass and lean mass will be performed.

At discharge, the children will be classified by the model of Cohen concerning medical complexities (CMC).^26^ A child will be classified as CMC when it meets all four of Cohen’s domains: having a chronic condition, high family needs, functional limitations and high healthcare use. The chronic condition domain will be met if the child was diagnosed with a complex chronic condition according to the model of Feudtner *et al*,^27^ and/or if there is at least 6 months continuous dependence on technology.^28^ The functional limitation domain will be met if patients were discharged with any of the following: tracheostomy, mechanical ventilation, oxygen therapy, airway clearance intervention techniques, monitoring of oxygen saturation, intravenous therapy medications (total parenteral nutrition, medication through central or peripherally inserted central catheter line), feeding tube (nasogastric tube, gastrostomy and jejunostomy) and colostomy. The domain high family needs were met if patients were discharged with more than three different types of medications and/or if more than two allied healthcare professionals kept involved in primary or outpatient care (rehabilitation physician, dietician, physiotherapist, occupational therapist and speech therapist). The domain for high healthcare use will be met calculating total days.

#### Harms

For details of assessment of harms, see online supplemental material A.

### Statistical analysis

All analyses will be performed according to the intention-to-treat principle. The intention-to-treat cohort is described by outlining patient flow, which will be presented using a CONSORT flow diagram adapted for SW-CRCTs, stratified by allocated sequence and period.^29 30^ The primary clinical endpoint is nutritional status, assessed through growth parameters (weight, length and head circumference). Secondary endpoints include feeding-related outcomes (nutritional intake, duration of nutritional support and feeding behaviour or problems), physical and cognitive development, emotional functioning, parental stress and body composition (fat mass and fat-free mass).

Baseline characteristics and treatments will be reported separately for the control (usual care) and intervention groups. Depending on data distribution, variables will be summarised as mean±SD, median with IQR (25th–75th percentile) or as frequencies and percentages. Missing data are expected mainly for secondary outcomes and questionnaire-based measures. For the primary outcome: nutritional status based on repeated growth measurements, missing values will be handled within the mixed-effects modelling framework, assuming data are missing at random. For secondary outcomes, multiple imputation using chained equations will be applied, taking the clustered study design into account. Analyses will be based on pooled imputed datasets. Sensitivity analyses will explore different assumptions about missingness, including both missing at random and missing not at random scenarios, with clustering incorporated. Figure 3 presents the directed acyclic graph underlying our covariate adjustment strategy, distinguishing design-induced confounders (calendar time, cluster) from prognostic covariates included to improve precision.^31 32^ The effect of the nutritional intervention compared with standard care will be analysed using mixed-effects regression models. Intervention status and calendar time will be included as fixed effects, and cluster (hospital) as a random effect to account for within-cluster correlation. Models will be adjusted for predefined confounders such as age, illness type and severity, nutritional status at admission and relevant nutritional risk scores.

**Figure 3:**
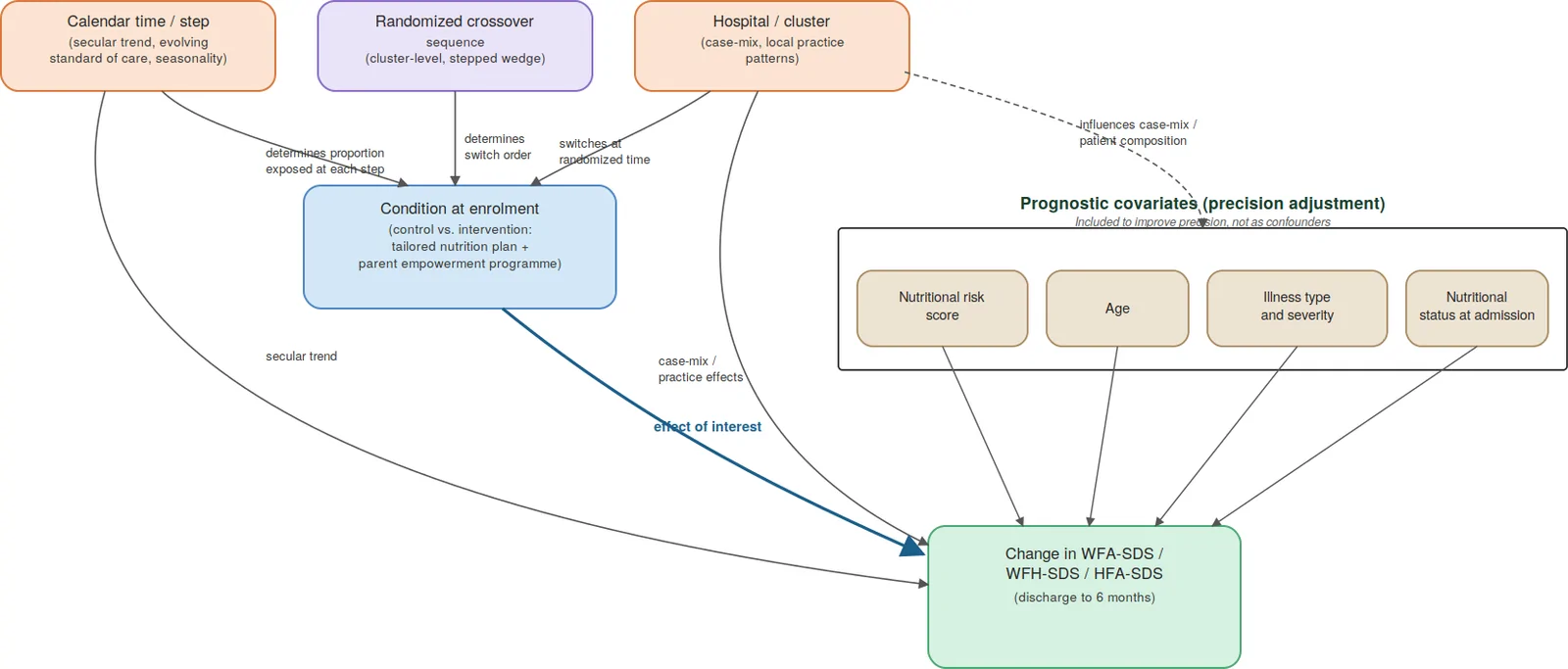
Directed acyclic graph. HFA-SDS, height‑for‑age SD‑score; WFA-SDS, weight-for-age SD-score; WFH-SDS, weight-for-height SD-score.

Discrete outcomes will be summarised as frequencies and percentages and analysed using χ² tests, Fisher’s exact tests or logistic regression, as appropriate. Continuous outcomes will be analysed using linear mixed-effects models; if assumptions are violated, transformations or non-parametric methods will be used. Time-to-event outcomes, such as time to discontinuation of nutritional supplementation, will be analysed using Cox proportional hazards models to compare timing and reasons for discontinuation between groups.

All analyses will be performed with and without adjustment for the predefined confounders. A two-sided p value ≤0.05 will be considered statistically significant. No correction for multiple testing will be applied due to the exploratory nature of the secondary outcomes.

#### Primary study parameter(s)

The change in growth (SD weight-for-age for children <1 year or weight-for-height for children ≥1 year) between the intervention group and the control group at the individual level will be compared corrected for the cluster-effect. Participants within a cluster might share characteristics and the care provided on the level of clusters might differ. The period effect, correlation within clusters and treatment heterogeneities will be assessed. Calculating a cluster-period (aggregated) measure is needed to be able to deal with treatment and period effects. The type of analyses will depend on whether there is a period effect. Generalised linear mixed models with treatment-period interaction terms will be assessed to check for randomness of interactions. Secondarily, models will be adjusted for possible confounders (eg, socio-economic status, baseline characteristics, clinical factors). When period effects are absent, meta-analysis methods with random effects will be used (quantify and test heterogeneity of the effect sizes within clusters). We will calculate the effect measure in each cluster and examine the consistency in effect across clusters, to be visualised (eg, Forest plots) and quantified (*τ*^2^ and *I*^2^ as heterogeneity measures).

#### Secondary study parameter(s)

Secondary outcomes will be compared between the intervention and the control group in a similar model to the one used in the analysis primary outcome.

### Data management and trial monitoring

For details of data management and trial monitoring, see online supplemental material A.

### Trial status

All six participating hospitals have commenced data collection. The first patient was enrolled on 23 April 2024, which corresponds with the registration date of this study. Final inclusion will be on 25 June 2026. Data collection is expected to continue until December 2026. The current protocol version reflects the amendments made after 9 months of enrolment to address slower-than-anticipated recruitment rates (as described earlier in this manuscript).

### Patient and public involvement

None.

## Ethics and dissemination

The protocol and informed consent forms were approved by the Institutional Review Board of Erasmus MC, Rotterdam (MEC-2023-0490). Formal ethical approval was waived at all other participating hospitals; each site obtained local Board of Directors authorisation. The study is conducted in accordance with the Declaration of Helsinki (2013) and the Dutch Medical Research Involving Human Subjects Act (WMO).

Informed consent: Consent procedures are differentiated by age per Dutch law: participants under 12 years—written consent from parents/legal guardians; 12–15 years—parental consent plus participant assent; 16 years and older—written consent from the participant. Examples participant information and consent forms are provided as online supplemental material B and online supplemental material C.

The NutriCAD trial is registered in the Overview of Medical Research in the Netherlands; onderzoekmetmensen.nl (OMON) under NL-NL-OMON56271 (registered 23 April 2024, prior to first patient enrolment). The OMON is operated by the Dutch Central Committee on Research Involving Human Subjects (CCMO) and is an International Committee of Medical Journal Editors (ICMJE) recognised registry linked to the WHO International Clinical Trials Registry Platform (ICTRP). A secondary ClinicalTrials.gov record exists (NCT06603792); per Erasmus MC policy, the OMON record serves as the primary registration.

Results will be disseminated through peer-reviewed publications and presentations at national and international scientific conferences, irrespective of direction or magnitude of findings. This is an investigator-initiated study; the funder had no role in study design, data collection, analysis, decision to publish or preparation of the manuscript. A plain-language summary will be made available via the trial registry on completion.

## Discussion

The NutriCAD trial addresses a clinically important and largely understudied gap in paediatric care: the structured nutritional management of children in the weeks and months following hospital discharge. While malnutrition in hospitalised children is well-recognised, the burden and consequences of ongoing nutritional support after discharge, including feeding disorders, parental stress and excessive weight gain, have received comparatively little research attention and no randomised trial has previously evaluated a structured, multidisciplinary follow-up intervention in this population.

The choice of a stepped-wedge cluster randomised design reflects both pragmatic and ethical considerations. Pragmatically, the design accommodates the reality that implementing a multidisciplinary nutrition support team is a cluster-level (hospital-level) intervention that cannot be applied to individual patients without contaminating the control condition. Ethically, the cross-over design ensures that all participating hospitals, and ultimately all enrolled patients, receive the intervention by the end of the study, which is appropriate given the anticipated benefit.

The breadth of the outcome measure set is a particular strength of this trial. By extending beyond conventional growth metrics to include body composition, dietary intake, feeding behaviour, parental psychological outcomes and a comprehensive economic evaluation, NutriCAD will generate evidence that is directly relevant to clinical decision-making, healthcare policy and health technology assessment. The inclusion of air displacement plethysmography (Pea Pod/Bod Pod) at the academic centre provides a uniquely rigorous gold-standard comparison for the more scalable measures (skinfolds, InBody BIA) used across all sites.

The study has several limitations. This study did not include formal patient and public involvement (PPI) in its design. The absence of lived-experience input from patients and families is a recognised limitation, particularly given the parent empowerment component and the nested qualitative implementation sub study. Future confirmatory work should incorporate structured PPI from the outset. Furthermore, the sample size calculation assumes that the effect of the intervention is homogeneous across patient subgroups (by age, diagnosis and level of care), which may not hold in practice. The amendment to the stepped-wedge schedule, adding two midstudy inclusion periods, partially mitigated the reduction in power caused by slower-than-anticipated recruitment, but the trial will be conducted at approximately 53% power, limiting its ability to detect a statistically significant effect of the anticipated magnitude. Findings should therefore be interpreted cautiously, particularly for secondary outcomes. Additionally, blinding of participants, families and clinicians is not feasible given the nature of the intervention, introducing a risk of performance and detection bias for subjective outcomes.

Notwithstanding these limitations, NutriCAD represents an important step towards establishing an evidence base for structured postdischarge nutritional care in paediatrics. If the intervention is shown to improve growth and nutritional outcomes, shorten the duration of nutritional support, reduce parental distress and be cost-effective, it has the potential to change practice and policy in the Netherlands and beyond.

## Supplementary material

10.1136/bmjopen-2026-122183 online supplemental file 1

10.1136/bmjopen-2026-122183 online supplemental file 2

10.1136/bmjopen-2026-122183 online supplemental file 3

10.1136/bmjopen-2026-122183 online supplemental file 4

## References

[1] Bélanger V, McCarthy A, Marcil V (2019). Assessment of Malnutrition Risk in Canadian Pediatric Hospitals: A Multicenter Prospective Cohort Study. J Pediatr.

[2] Hulst JM, Zwart H, Hop WC (2010). Dutch national survey to test the STRONGkids nutritional risk screening tool in hospitalized children. *Clin Nutr*.

[3] Joosten KF, Zwart H, Hop WC (2010). National malnutrition screening days in hospitalised children in The Netherlands. Arch Dis Child.

[4] Werner D, Zimmer K-P, Eckert GP (2020). Malnutrition in Paediatrics: Stationary Screening & Follow-up. *Klin Padiatr*.

[5] Black MM, Dubowitz H, Krishnakumar A (2007). Early intervention and recovery among children with failure to thrive: follow-up at age 8. Pediatrics.

[6] Corbett SS, Drewett RF (2004). To what extent is failure to thrive in infancy associated with poorer cognitive development? A review and meta-analysis. *J Child Psychol Psychiatry*.

[7] Galler JR, Ramsey F, Solimano G (1983). The Influence of Early Malnutrition on Subsequent Behavioral Development: I. Degree of Impairment in Intellectual Performance. J Am Acad Child Psychiatry.

[8] Hecht C, Weber M, Grote V (2015). Disease associated malnutrition correlates with length of hospital stay in children. Clin Nutr.

[9] Hulst J, Joosten K, Zimmermann L (2004). Malnutrition in critically ill children: from admission to 6 months after discharge. *Clin Nutr*.

[10] Medoff-Cooper B, Irving SY, Hanlon AL (2016). The Association among Feeding Mode, Growth, and Developmental Outcomes in Infants with Complex Congenital Heart Disease at 6 and 12 Months of Age. J Pediatr.

[11] Ravishankar C, Zak V, Williams IA (2013). Association of impaired linear growth and worse neurodevelopmental outcome in infants with single ventricle physiology: a report from the pediatric heart network infant single ventricle trial. J Pediatr.

[12] Marino LV, Collaço NC, Ashton JJ (2022). Pedi-R-MAPP: The development of a nutritional awareness tool for use in remote paediatric consultations using a modified Delphi consensus. Clin Nutr.

[13] Diamanti A, Cereda E, Capriati T (2019). Prevalence and outcome of malnutrition in pediatric patients with chronic diseases: Focus on the settings of care. Clin Nutr.

[14] Krom H, van Zundert SMC, Otten M-A (2019). Prevalence and side effects of pediatric home tube feeding. Clin Nutr.

[15] Kooij M, Koster E, Eveleens R (2023). Nutritional follow-up in children after discharge: Organisation in a tertiary care centre. J Hum Nutr Diet.

[16] Senez C, Guys JM, Mancini J (1996). Weaning children from tube to oral feeding. *Child's Nerv Syst*.

[17] Brinksma A, Sulkers E, IJpma I (2020). Eating and feeding problems in children with cancer: Prevalence, related factors, and consequences. Clin Nutr.

[18] Colville G, Pierce C (2012). Patterns of post-traumatic stress symptoms in families after paediatric intensive care. Intensive Care Med.

[19] Lutz KF (2012). Feeding Problems of NICU and PICU Graduates: Perceptions of Parents and Providers. Newborn Infant Nurs Rev.

[20] Wells JC, Sawaya AL, Wibaek R (2020). The double burden of malnutrition: aetiological pathways and consequences for health. The Lancet.

[21] Woo Baidal JA, Locks LM, Cheng ER (2016). Risk Factors for Childhood Obesity in the First 1,000 Days. Am J Prev Med.

[22] Hemming K, Taljaard M, McKenzie JE (2018). Reporting of stepped wedge cluster randomised trials: extension of the CONSORT 2010 statement with explanation and elaboration. BMJ.

[23] Addo OY, Himes JH, Zemel BS (2017). Reference ranges for midupper arm circumference, upper arm muscle area, and upper arm fat area in US children and adolescents aged 1–20 y. Am J Clin Nutr.

[24] WHO (2013). Utilization of mid-upper arm circumference versus weight-for-height in nutritional rehabilitation programmes: a systematic review of evidence.

[25] WHO O (2009). WHO child growth standards and the identification of severe acute malnutrition in infants and children: a joint statement by the world health organization and the united nations children’s fund.

[26] Rowen D, Mulhern B, Stevens K (2018). Estimating a Dutch Value Set for the Pediatric Preference-Based CHU9D Using a Discrete Choice Experiment with Duration. Value in Health.

[27] Feudtner C, Feinstein JA, Zhong W (2014). Pediatric complex chronic conditions classification system version 2: updated for ICD-10 and complex medical technology dependence and transplantation. BMC Pediatr.

[28] Popalisky J, Lyons D, Woodcox P (2014). Center of Excellence on Quality of Care Measures for Children with Complex Needs Medical Complexity Working G. Pediatric medical complexity algorithm: a new method to stratify children by medical complexity. Pediatrics.

[29] Hemming K, Haines TP, Chilton PJ (2015). The stepped wedge cluster randomised trial: rationale, design, analysis, and reporting. BMJ.

[30] Hussey MA, Hughes JP (2007). Design and analysis of stepped wedge cluster randomized trials. Contemp Clin Trials.

[31] Rubin D (2026). Adjusting for covariates in randomized clinical trials for drugs and biological products. Clinical Trials.

[32] Tsiatis AA, Davidian M, Zhang M (2008). Covariate adjustment for two-sample treatment comparisons in randomized clinical trials: a principled yet flexible approach. Stat Med.

[33] Zemel BS, Riley EM, Stallings VA (1997). EVALUATION OF METHODOLOGY FOR NUTRITIONAL ASSESSMENT IN CHILDREN: Anthropometry, Body Composition, and Energy Expenditure. Annu Rev Nutr.

[34] M. Marfell-Jones ADS JHdR (2012). International standards for anthropometric assessment.

[35] Nguyen QD, Fusch G, Armbrust S (2007). Impedance index or standard anthropometric measurements, which is the better variable for predicting fat-free mass in sick children?. Acta Paediatr.

[36] Rosendale RP, Bartok CJ (2012). Air-displacement plethysmography for the measurement of body composition in children aged 6–48 months. Pediatr Res.

[37] Fields DA, Goran MI, McCrory MA (2002). Body-composition assessment via air-displacement plethysmography in adults and children: a review. Am J Clin Nutr.

[38] Gezondheidsraad DN (2022). Voedingsnormen voor energie.

[39] Gezondheidsraad DN (2021). Voedingsnormen voor eiwitten.

[40] Netherlands. HCot (2025). Dietary reference values for vitamins and minerals for infants and children.

[41] ondervoeding K (2022). Screening op en behandeling van ondervoeding bij kinderen opgenomen in nederlandse ziekenhuizen.

[42] Ramsay M, Martel C, Porporino M (2011). The Montreal Children’s Hospital Feeding Scale: A brief bilingual screening tool for identifying feeding problems. Paediatr Child Health.

[43] Llewellyn CH, van Jaarsveld CHM, Johnson L (2011). Development and factor structure of the Baby Eating Behaviour Questionnaire in the Gemini birth cohort. Appetite.

[44] Wardle J, Guthrie CA, Sanderson S (2001). Development of the Children’s Eating Behaviour Questionnaire. *Child Psychology Psychiatry*.

[45] Haverman L, van Oers HA, Limperg PF (2013). Development and validation of the distress thermometer for parents of a chronically ill child. J Pediatr.

[46] Jongedijk RA, Olff M (2014). PTSD Checklist for DSM-5– Nederlandstalige Versie. Diemen: Stichting Centrum ’45.

[47] Terwee CB, Roorda LD, de Vet HCW (2014). Dutch-Flemish translation of 17 item banks from the patient-reported outcomes measurement information system (PROMIS). Qual Life Res.

[48] Zorg PU (2022). Adviesrapport set generieke PRO(M)s.

[49] Mauskopf JA, Sullivan SD, Annemans L (2007). Principles of Good Practice for Budget Impact Analysis: Report of the ISPOR Task Force on Good Research Practices—Budget Impact Analysis. Value in Health.

[50] Stevens K (2009). Developing a descriptive system for a new preference-based measure of health-related quality of life for children. Qual Life Res.

[51] Stevens K (2012). Valuation of the Child Health Utility 9D Index. Pharmacoeconomics.

[52] Institute for Medical Technology Assessment EUR (2013). IMTA medical consumption questionnaire.

[53] Technology Assessment EUR (2015). The imta productivity cost questionnaire: a standardized instrument for measuring and valuing health-related productivity losses.

