# Supplementary material for: Nutritional Care After Hospital Discharge (NutriCAD): protocol for a stepped-wedge, multicentre, randomised controlled trial in paediatrics
Source: BMJ Open. 2026 Oct 27;16(10):e122183. doi: 10.1136/bmjopen-2026-122183 (PMC13723836; doi:10.1136/bmjopen-2026-122183)
Supplement: Supplementary file 1 [file bmjopen-16-10-s002.pdf]

Informatie Formulier 16 jaar en ouder

Voedingszorg na ontslag

Nutritional Care After Discharge (nutriCAD)

Inleiding

Beste,

Met deze informatiebrief willen we vragen of je wilt meedoen aan medisch-wetenschappelijk onderzoek. Meedoen is vrijwillig. Je krijgt deze brief omdat je naar huis gaat met voedingszorg of voedingsadviezen.
Je leest hier om wat voor onderzoek het gaat, wat het voor jou betekent, en wat de voordelen en nadelen zijn. Het is veel informatie. Wil je deze informatie doorlezen en beslissen of je wilt meedoen? Als je wilt meedoen, kan je het formulier invullen dat te vinden is in bijlage B.

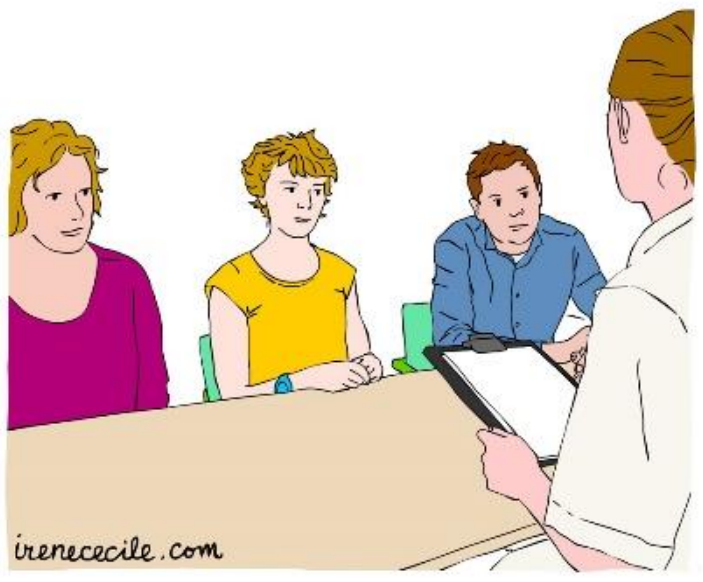

Stel je vragen

Je kunt je beslissing nemen met de informatie die je in deze informatiebrief vindt.
Daarnaast raden we je aan om dit te doen:
- Stel vragen aan de onderzoeker die je deze informatie geeft.
- Praat met je familie of vrienden over dit onderzoek.
- Stel vragen aan de onafhankelijk deskundige. Voor contactgegevens zie bijlage A.
- Lees de informatie op www.rijksoverheid.nl/mensenonderzoek of https://www.kindenonderzoek.nl/

1. Algemene informatie

Dit onderzoek wordt uitgevoerd in het Erasmus MC Sophia kinderziekenhuis, maar ook in andere ziekenhuizen in Nederland. Er doen in totaal 6 ziekenhuizen mee aan dit onderzoek en we willen dat er ongeveer 260 kinderen en/of jongeren meedoen. Het onderzoek is gecontroleerd door een speciaal team, de Medisch Ethische Toetsingscommissie van het Erasmus MC. Zij hebben beoordeeld dat dit onderzoek nagenoeg niet belastend is.

2. Wat is het doel van het onderzoek

In het onderzoek willen we uitzoeken of een op maat gemaakt voedingsplan in combinatie met ondersteuning van je ouder(s)/verzorger(s) een positief effect heeft op verschillende factoren. We kijken dan naar je groei en lichaamssamenstelling. Ook bekijken we je voedingsinname en voedingsgedrag. Verder zullen we onderzoek doen naar het verminderen van stress bij je ouders(s)/verzorger(s) en verbetering op emotioneel welbevinden. Als laatste willen we kijken of het op maat gemaakte voedingsplan effectiever is en minder kost dan de huidige zorg.

3. Achtergrond van het onderzoek

Het is belangrijk voor je groei en ontwikkeling dat je na je opname uit het ziekenhuis goed herstelt en weer aansterkt. Daarom ga je naar huis met voedingszorg. Voedingszorg kan zijn dat je adviezen hebt gekregen om meer of anders te eten, je kan dieetpreparaten hebben gekregen zoals drinkvoeding, of het kan zijn dat je met sondevoeding naar huis gaat. Hiervoor krijg je begeleiding van de kinderarts en/of diëtist. Maar we weten nog niet welke manier van zorg het beste is zodat je niet langer dan nodig de voedingsadviezen hoeft op te volgen. Dit willen we graag onderzoeken. Daarnaast kan het ook spannend of stressvol zijn om naar huis te gaan met voedingsadviezen en kan dit van invloed zijn op jouw leven en dat van je ouder(s)/verzorger(s). Hier weten wij nog niet veel over en daarom willen we dit onderzoeken.

4. Hoe verloopt het onderzoek

Om het effect te kunnen onderzoeken van een op maat gemaakt voedingsplan met ondersteuning van je ouder(s)/verzorger(s) zullen er twee groepen gemaakt worden:

1) Controlegroep: deze patiënten krijgen geen aanpassing in de zorg, maar krijgen de standaard voedingszorg. We willen echter informatie verzamelen over hoe dat gaat. Bij punt 5 kan je lezen wat we dan precies van jou verwachten.
2) Interventiegroep: deze patiënten krijgen een aanpassing in de standaardzorg. Hierbij krijg je gestructureerde follow-up afspraken bij de diëtist. Voor deze afspraken wordt vooraf informatie verzameld. We willen jou en je ouders vragen deze informatie in te vullen. Aan de hand van deze informatie ontvang je een voedingsadvies op maat. Wat je dan precies allemaal gaat doen is te lezen onder punt 5.

Nadat je toestemming hebt gegeven om mee te doen aan het onderzoek zal bekend worden in welke groep je zit. Het vragen om deel te nemen aan de studie en het geven van toestemming heet includeren.

Hoe weet je in welke groep je zit?

Er wordt niet individueel per patiënt bepaald in welke groep je zit, maar dit is afhankelijk van het moment waarop je wordt geïncludeerd in het ziekenhuis. We loten namelijk vooraf wanneer jouw ziekenhuis zal starten met het includeren van de interventiegroep.

Alle ziekenhuizen beginnen met het verzamelen van informatie over hoe de standaardzorg gaat, de controlegroep. Vervolgens start er elke 3 maanden één ziekenhuis met de inclusie van patiënten in de interventiegroep. En het ziekenhuis zal dit blijven doen tot het einde van de studie. Bijvoorbeeld: ziekenhuis A begint in januari met de inclusie van de inventiegroep en de patiënten die mee mogen en willen doen krijgen de interventie. De andere ziekenhuizen verzamelen op hetzelfde moment nog steeds informatie over de standaardzorg. Ziekenhuis B start in april met het includeren

van de interventiegroep, enzovoort. We weten dus van tevoren in welke groep je zit. We vertellen dit liever nog niet voordat j toestemming hebt gegeven om mee te doen. Het kan namelijk de informatie die we verzamelen beïnvloeden. Je kan namelijk erg gemotiveerd zijn om mee te doen als je weet in welke groep je valt, of juist minder gemotiveerd. Dit willen we voorkomen. Als je niet deelneemt aan de studie krijgt je altijd de standaardzorg, zonder het verzamelen van de informatie in studieverband.

Wat is er anders dan bij gewone zorg?

Als je in de controlegroep zit is er niet zoveel anders dan bij gewone zorg. We willen echter wel wat informatie verzamelen. Dit doen we bij ontslag uit het ziekenhuis en we vragen jou dan om 6 maanden na ontslag voor een studiebezoek te komen. Tussentijds sturen we een vragenlijst op om informatie te verzamelen over de gebruikte zorg. In paragraaf 5 kan je meer lezen over wat er specifiek verzameld wordt.
Als je in de interventiegroep zit is het verschil dat er op vaste tijdstippen follow-up momenten gepland staan waarbij vooraf informatie wordt verzameld over jouw voeding, groei en eetgedrag. Er wordt ook gekeken naar stress bij je ouder(s)/verzorger(s). Dit wordt gebruikt om met een multidisciplinair team een voedingsplan op maat te maken.

5. Welke afspraken maken we met jou?

Als je toestemming hebt gegeven volgt er een ontslaggesprek met de (onderzoeks)dietist. Dan wordt ook duidelijk in welke groep je komt en worden de volgende afspraken gemaakt:

1. Controlegroep: we zien je graag terug 6 maanden na ontslag uit het ziekenhuis. Voor de tussenliggende periode krijgen jij en je ouder(s)/verzorger(s) een logboek mee om wat gegevens in te vullen. Daarnaast zal er tussentijds een vragenlijst gestuurd worden via de mail om de gemaakte (zorg)kosten in kaart te brengen.
2. Interventiegroep: we zien jou en je ouder(s)/verzorger(s) graag terug voor de studie: rond 6, 12 en 18 weken na ontslag . Dit zijn afspraken die eventueel ook gecombineerd kunnen worden met andere afspraken die je die dag hebt. Indien het nodig is voor jouw zorg kunnen tussentijdse (telefonische) afspraken gewoon nog ingepland worden naast de studiebezoeken. Maar hiervan worden geen gegevens verzameld voor de studie. Verder willen we je nog een keer terug zien 6 maanden na ontslag uit het ziekenhuis.

Metingen en vragenlijsten

Voor elk studiebezoek willen we jou en je ouder(s)/verzorger(s) vragen en aantal dingen te doen:

- Een 3-daags voedingsdagboek bijhouden
- Een aantal vragenlijsten invullen

Tijdens de studiebezoeken willen we een aantal metingen doen:

- Gewicht, lengte en bovenarmomtrek
- Lichaamssamenstelling metingen. Om dit te meten gebruiken we verschillende methodes: De huidplooi, bio-elektrische impedantie analyse (BIA) en in het Sophia kinderziekenhuis hebben we ook nog een ander apparaat waarmee we dit kunnen meten namelijk de BODPOD. Alle drie de metingen doen geen pijn. Je kunt in onderstaande tabel lezen wat deze metingen inhouden.

**Meting**	**Uitleg**
**Huidplooi**	
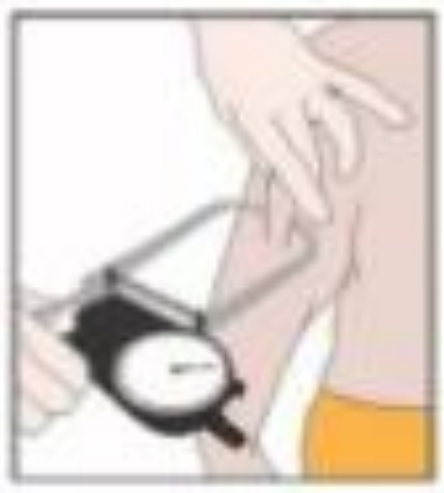	Met de huidplooimeter kan de dikte van een huidplooi worden bepaald. Met behulp van de gemeten huidplooien kan een voorspelling worden gedaan over de totale lichaamsvetmassa. Met behulp van referentietabellen kan een schatting gemaakt worden van de vetmassa en vetvrije massa van het lichaam.
**Bio-elektrische impedantie (BIA)**	
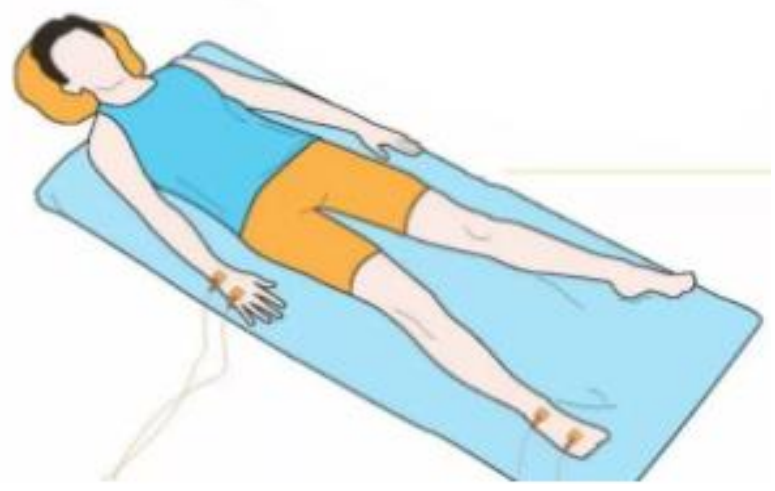	Bij de BIA wordt de impedantie (samengesteld uit de weerstand en schijnweerstand) gemeten van een wisselstroom die door een lichaam wordt gestuurd. Dit gebeurt door middel van elektroden die worden geplaatst op de hand en voet. Door middel van formules wordt de lichaamssamenstelling bepaald.
**BODPOD**	
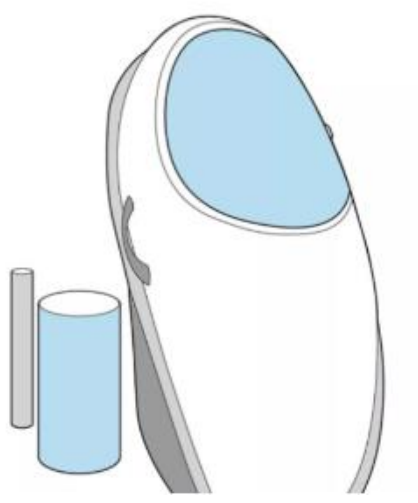	Een BODPOD is een cabine die verschillen in luchtdruk meet, nadat iemand in de capsule gaat zitten. De BODPOD bepaalt binnen enkele minuten het volume van het lichaam en kan vervolgens door middel van formules de lichaamssamenstelling bepalen

We zullen alle verzamelde gegevens direct gebruiken in het voedingsadvies dat we samen met je opstellen. En de metingen die we doen gebruiken we om te evalueren of de doelen die we als onderzoekers gesteld hebben ook gehaald worden.

6. Van welke bijwerkingen, nadelige effecten of ongemakken kan je last krijgen

Er zullen géén vervelende/pijnlijke dingen gebeuren voor het onderzoek, zoals bloedprikken.

7. Wat zijn de voordelen en nadelen als je meedoet aan het onderzoek?

- Als je in de controlegroep wordt ingeloot zal er niks veranderen aan de zorg, maar help je mee aan wetenschappelijk onderzoek, en help je daardoor de zorg telkens een beetje beter te maken.
- 6 maanden na het ontslag uit het ziekenhuis gaan we kijken hoe je groeit, wat je eet en hoe de lichaamssamenstelling is van jou.
- Een nadeel is dat je een extra bezoekje moet brengen aan het ziekenhuis 6 maanden na ontslag uit het ziekenhuis.
- Als je in de interventiegroep wordt ingeloot kan het gunstiger zijn voor je groei, stress bij je ouders en de tijd dat je de voedingsadviezen moet opvolgen. Maar dat weten we nog niet.
- Als je in de interventiegroep komt moeten je ouder(s)/verzorger(s) vragenlijsten invullen en zullen daar tijd aan kwijt zijn.

Wil je niet meedoen?

Jij beslist zelf of je meedoet aan het onderzoek. Wil je niet meedoen? Dan krijg je de gewone voedingszorg zoals dat in jouw ziekenhuis geregeld is.

8. Wanneer stopt het onderzoek?

De onderzoeker laat het je weten als er nieuwe informatie over het onderzoek naar voren komt die belangrijk voor je is. De onderzoeker vraagt daarna of je blijft meedoen.

Jouw deelname aan het onderzoekt stopt als:

- Alle studiebezoeken zoals beschreven staat onder ''bezoeken'' voorbij zijn. Dit is 6 maanden na je ontslag uit het ziekenhuis, de laatste poli afspraak.
- Jij zelf ervoor kiest om te stoppen met het onderzoek. Dat mag op ieder moment. Meld dit dan meteen bij de onderzoeker. Je hoeft er niet bij te vertellen waarom je stopt. Je krijgt dan weer de gewone voedingszorg.
- De onderzoeker het beter vindt om te stoppen.

Wat gebeurt er als je stopt met het onderzoek?

De onderzoekers gebruiken de gegevens die tot het moment van stoppen zijn verzameld. Het hele onderzoek is afgelopen als alle deelnemers klaar zijn.

9. Wat gebeurt er na het onderzoek?

Het hele onderzoek is afgelopen als alle deelnemers klaar zijn. Maar na 6 maanden stopt het onderzoek voor jou en zal indien nodig de voedingszorg vervolgd worden bij de diëtist in het ziekenhuis waar jij ook bij kinderarts op afspraak komt. Na het verwerken

van alle gegevens zal de onderzoeker je op de hoogte brengen van de belangrijkste uitkomsten van het onderzoek. Het kan zijn dat dit pas na een paar jaar bekend wordt. De informatie gaat over de hele studie en niet over jouw persoonlijke resultaten.

10. Wat doen we met jouw gegevens

Doe je mee met het onderzoek? Dan geef je ook toestemming om jouw gegevens te verzamelen, gebruiken en bewaren.

Welke gegevens bewaren we?

Persoonsgegevens = informatie over wie jij bent, bijvoorbeeld je geboortedatum en waar je woont.	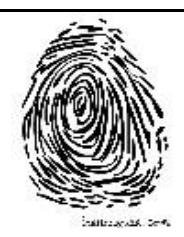
Medische gegevens = (ook een soort persoonsgegevens) informatie over je gezondheid, bijvoorbeeld of je ziek bent en of je medicijnen gebruikt.	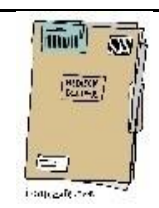
Groei gegevens en lichaamssamenstelling = We zullen je lengte en gewicht meten tijdens de bezoeken aan het ziekenhuis. Maar ook zullen we meten met een apparaat hoe jouw lichaam is opgebouwd. Dan kijken we hoeveel spiermassa je hebt.	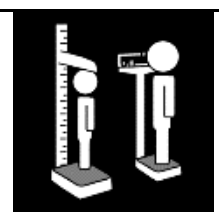
Voedingsgegevens = We zullen berekenen en verzamelen wat je aan voeding nodig hebt en wat je gegeten hebt.	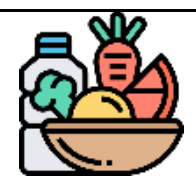
Vragenlijsten = we zullen vragen aan je ouder(s)/verzorger(s) of aan jouzelf om een aantal vragenlijsten in te vullen over stress en kwaliteit van leven als jij voedingszorg krijgt	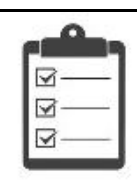

Waarom verzamelen, gebruiken en bewaren we je gegevens?

We verzamelen, gebruiken en bewaren je gegevens om de vragen van dit onderzoek te kunnen beantwoorden. En om de resultaten te kunnen publiceren.

Hoe beschermen we je privacy?

Om je privacy te beschermen geven wij je gegevens een code. Op al je gegevens zetten we alleen deze code. De sleutel van de code bewaren we op een beveiligde plek in het ziekenhuis. Als we je gegevens verwerken, gebruiken we steeds alleen die code. Ook in rapporten en publicaties over het onderzoek kan niemand terughalen dat het over jou ging.

Wie kunnen je gegevens zien?

Sommige personen kunnen wel je naam en andere persoonlijke gegevens zonder code inzien. Dit kunnen gegevens zijn die speciaal voor dit onderzoek zijn verzameld, maar ook gegevens uit je medisch dossier.

- Dit zijn mensen die controleren of de onderzoekers het onderzoek goed en betrouwbaar uitvoeren: Leden van de commissie die de veiligheid van het onderzoek in de gaten houdt.
- Een controleur die voor het ziekenhuis werkt.

Deze personen houden je gegevens geheim. Voor inzage door deze personen vragen wij je toestemming te geven. De Inspectie Gezondheidszorg en Jeugd kan zonder jouw toestemming je gegevens inzien.

Hoelang bewaren we je gegevens?

We bewaren je gegevens 10 jaar in het ziekenhuis.

Kan je je toestemming voor het gebruik van je gegevens weer intrekken?

Je kunt je toestemming voor het gebruik van je gegevens op ieder moment intrekken. Zeg dat dan tegen de onderzoeker. Dit geldt voor het gebruik in dit onderzoek en voor het gebruik in ander onderzoek. Maar let op: trek je je toestemming in en hebben onderzoekers dan al gegevens verzameld? Dan mogen ze deze gegevens nog wel gebruiken.

Wil je meer weten over je privacy?

- Wil je meer weten over je rechten bij de verwerking van persoonsgegevens? Kijk dan op www.autoriteitpersoonsgegevens.nl.
- Heb je nog vragen over je rechten? Of heb je een klacht over de verwerking van je persoonsgegevens? Neem dan contact op met degene die verantwoordelijk is voor de verwerking van je persoonsgegevens. Voor dit onderzoek is dat:
  - ErasmusMC Sophia kinderziekenhuis. Zie bijlage A voor contactgegevens, en website.
  - Als je klachten hebt over de verwerking van je persoonsgegevens, raden we aan om deze eerst te bespreken met het onderzoeksteam. Je kunt ook naar de Functionaris Gegevensbescherming van je ziekenhuis gaan. Of je dient een klacht in bij de Autoriteit Persoonsgegevens.

11. Krijg je een vergoeding als je meedoet aan het onderzoek?

Je krijgt geen vergoeding als je meedoet aan dit onderzoek. Wel krijg je een vergoeding voor de (extra) reiskosten

12. Ben je verzekerd tijdens het onderzoek?

Je bent niet extra verzekerd voor dit onderzoek. Want meedoen aan het onderzoek heeft geen extra risico's. Daarom hoeft het Erasmus MC van de METC geen extra verzekering af te sluiten.

13. We informeren huisarts en/of behandelend specialist

De onderzoeker stuurt je huisarts en/of behandelend specialist een brief om te laten weten dat je meedoet aan het onderzoek. Dit is voor jouw eigen veiligheid.

14. Heb je vragen?

Vragen over het onderzoek kun je stellen aan de onderzoeker. Wil je advies van iemand die er geen belang bij heeft? Ga dan naar de onafhankelijk deskundige, voor contactgegevens zie bijlage A. Zij weet veel over het onderzoek, maar werkt niet mee aan dit onderzoek.
Heb je een klacht? Bespreek dit dan met de onderzoeker of de arts die jou behandelt. Wil je dit liever niet? Ga dan naar klachtenfunctionaris/klachtencommissie van je ziekenhuis. In bijlage A staat waar je die kunt vinden.

15. Hoe geeft je toestemming voor het onderzoek?

Je kunt eerst rustig nadenken over dit onderzoek. Daarna vertel je de onderzoeker of je de informatie begrijpt en of je wel of niet wilt meedoen. Wil je meedoen, dan zet je een handtekening op het toestemmingsformulier. Ook kun je kiezen of je we je later mogen vragen voor een mogelijk volgend onderzoek. We geven je later informatie over een mogelijk nieuw onderzoek, daarna kun je beslissen of je wilt meedoen. Jij en de onderzoeker krijgen allebei een getekende versie van deze toestemmingsverklaring.

Bijlage A: Contactgegevens

Hoofdonderzoeker:

Dr. S.C.A.T Verbruggen
Afdeling IC neonatologie & kinderen, Sophia kinderziekenhuis
ErasmusMC medisch centrum, Rotterdam
s.verbruggen@erasmusmc.nl
010-7036044

Onafhankelijk onderzoeker

Dr. B.A.E de Koning
Afdeling kindergeneeskunde, kindermaag- darm- en leverziekten
ErasmusMC medisch centrum, Rotterdam

Onderzoeker

E.J van Steenbergen
Afdeling IC neonatologie & kinderen, Sophia kinderziekenhuis
ErasmusMC medisch centrum, Rotterdam
Nutricad@erasmusmc.nl

Klachten:

Indien je niet tevreden bent over het onderzoek of de behandeling, kun je terecht bij de onafhankelijke klachtenopvang/klachtenfunctionaris van het Erasmus MC.
Op de website van het Erasmus MC is een Digitaal klachtenformulier beschikbaar via https://www.erasmusmc.nl/nl-nl/patientenzorg/klachtenopvang-en-klachtenbemiddeling
Na het invullen wordt het formulier automatisch verzonden naar de klachtenfunctionaris.
Als het niet lukt om het digitale klachtenformulier in te vullen, dan kunt je je klacht ook per post versturen: Erasmus MC, Secretariaat Klachtenopvang (GK-745), Antwoordnummer 55, 3000 WB Rotterdam.
Vermeld in de brief je naam, patiëntnummer (indien van toepassing), naam van het onderzoek en contactgegevens. Na ontvangst van de brief zal de klachtenfunctionaris contact met je opnemen.

Functionaris voor de Gegevensbescherming van de instelling:

De Functionaris voor de Gegevensbescherming van het Erasmus MC is bereikbaar via het secretariaat van de afdeling Juridische Zaken. E-mail: functionaris.gegevensbescherming@erasmusmc.nl Tel: 010-703 4986
Voor meer informatie over uw rechten:

Voor meer informatie of bij vragen over je rechten kan je contact opnemen met de functionaris voor de Gegevensbescherming of met de Autoriteit Persoonsgegevens.

Bijlage B: Toestemmingsformulier voor kinderen van 16 jaar en ouder

Onderzoek naar voedingszorg na ontslag uit het ziekenhuis (NutriCAD) bij kinderen

- Ik heb de informatie **begrepen**. Ook kon ik vragen stellen. Mijn **vragen zijn beantwoord**.
- Ik had **genoeg tijd om te beslissen** of ik meedoe.
- Ik weet dat ik **niet verplicht** ben om mee te doen.
- Ik begrijp dat ik **altijd mag stoppen** als ik niet meer mee wil doen.
- Ik geef toestemming **voor het informeren** van mijn huisarts en specialist(en) die mij behandelt dat ik meedoe aan dit onderzoek

Kruis jouw antwoord aan

Ik geef toestemming voor het gebruik van gegevens uit mijn patiëntendossier voor dit onderzoek<br><br>*(Als je hier kiest voor 'nee', dan kun je niet meedoen aan dit onderzoek)*	☐ Ja<br>☐ Nee
Ik geef toestemming om mij later te vragen voor vervolgonderzoek	☐ Ja<br>☐ Nee
Mailadres voor de vragenlijst	
Mailadres ouders voor de vragenlijst	

Ik wil meedoen aan dit onderzoek.

Naam deelnemer:

Handtekening: Datum : __ / __ / __

<u>Dit stuk is voor de onderzoeker:</u>

Ik verklaar dat ik deze proefpersoon volledig heb geïnformeerd over het genoemde onderzoek. Als er tijdens het onderzoek informatie bekend wordt die de toestemming van de proefpersoon zou kunnen beïnvloeden, dan breng ik hem/haar daarvan tijdig op de hoogte.

Naam onderzoeker (of diens vertegenwoordiger):

Handtekening: Datum: __ / __ / __

<*indien van toepassing*> Aanvullende informatie is gegeven door:

Naam:

Functie:

Handtekening: Datum: __ / __ / __

De proefpersoon krijgt een volledige informatiebrief mee, samen met een getekende versie van het toestemmingsformulier.
