# Supplementary material for: Nutritional Care After Hospital Discharge (NutriCAD): protocol for a stepped-wedge, multicentre, randomised controlled trial in paediatrics
Source: BMJ Open. 2026 Oct 27;16(10):e122183. doi: 10.1136/bmjopen-2026-122183 (PMC13723836; doi:10.1136/bmjopen-2026-122183)
Supplement: Supplementary file 2 [file bmjopen-16-10-s003.pdf]

Informatie Formulier 12-15 jaar
Nutritional Care After Discharge

Hoi!

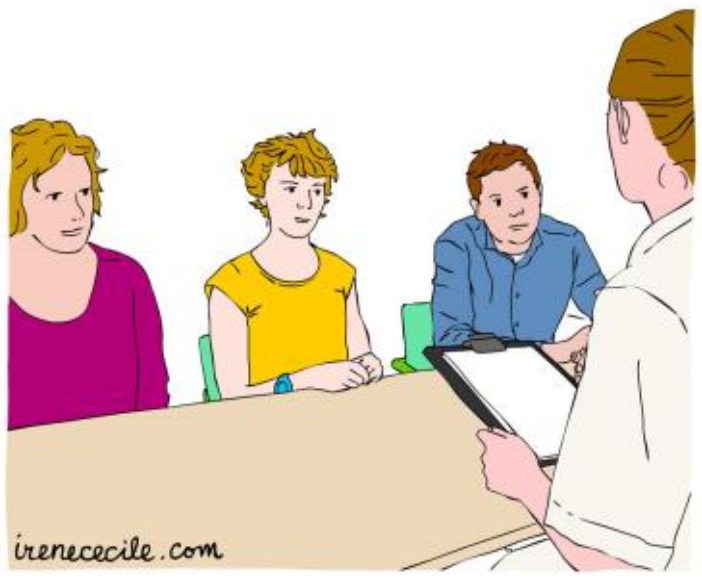

Doe je mee aan een onderzoek? Hier lees je meer over het onderzoek en jouw rechten. Lees dit goed, want dan weet je waarover je kunt beslissen. Je mag rustig nadenken voordat je beslist.
Je ouder(s)/verzorger(s) krijgen ook informatie over dit onderzoek. Je kunt samen met hen praten over het onderzoek. Zij zullen samen met jou een beslissing nemen.

Vragen en contact

Heb je vragen? Bespreek ze met je ouders. Of stel ze aan de onderzoeker. Je kunt je vragen hieronder opschrijven.

Je mag de onderzoeker ook altijd mailen: Esther van Steenbergen, nutricad@erasmusmc.nl

Wil je praten over het onderzoek met een arts die er niet bij betrokken is? Mail dan met: dr. B.A.E. de Koning

Ruimte om jouw vragen op te schrijven:

Tip: neem een foto van je vragen, dan heb je ze bij je als je met de arts/onderzoeker gaat praten.

Over het onderzoek

Dit onderzoek wordt gedaan in het Erasmus MC Sophia kinderziekenhuis, maar ook in andere ziekenhuizen in Nederland. Er doen in totaal 6 ziekenhuizen mee aan dit onderzoek en we willen dat er ongeveer 260 kinderen en/of jongeren meedoen. Het onderzoek is gecheckt door een speciaal team, de Medisch Ethische Toetsingscommissie van het Erasmus MC. Hun conclusie was dat dit onderzoek nagenoeg niet belastend is.

Waarom dit onderzoek?

Het is belangrijk dat je goed groeit. Als je het ziekenhuis verlaat, krijg je voedingsadvies om te zorgen dat je weer kan aansterken. Deze krijg je van de kinderarts en/of diëtist. We weten alleen nog niet goed welke zorg precies nodig is zodat je goed en snel kan aansterken. Daarnaast kan het ook spannend of stressvol zijn om naar huis te gaan met voedingsadviezen en kan dit van invloed zijn op jouw leven en dat van je ouder(s)/verzorger(s). Hier weten wij ook nog niet veel over en daarom willen we dit onderzoeken.

Achtergrond

We willen onderzoeken of extra voedingsadvies in combinatie met ondersteuning van je ouder(s)/verzorger(s) positief is voor je groei en spieren.
We zullen bij je ouders(s)/verzorger(s) ook kijken of dit plan stress kan verminderen.

Hoe werkt meedoen?

Er zijn twee verschillende groepen die we onderzoeken. Dit zijn:

1) Controlegroep: dan is er in jouw zorg geen aanpassing gemaakt, maar krijg je de standaard voedingszorg. We willen wel informatie verzamelen hoe dat nu gaat. Bij “over de behandeling” kan je lezen wat we dan precies gaan verzamelen.
2) Interventiegroep: dan is er een aanpassing in de standaard zorg. Hierbij krijg je op vaste tijdstippen follow-up afspraken bij de diëtist. Voor deze afspraken wordt vooraf informatie verzameld. We willen jou en je ouders vragen deze informatie in te vullen. Aan de hand van deze informatie ontvang je een voedingsadvies op maat. Bij “over de behandeling” kan je lezen wat dit allemaal precies is.

Nadat jij en je ouders toestemming hebben gegeven om mee te doen aan het onderzoek, zal er bekend gemaakt worden in welke groep jij zit. Het vragen om deel te nemen aan de studie en het geven van toestemming heet includeren.

Hoe weet je in welke groep je zit?

Dat wordt niet per kind bepaald maar ligt aan het moment waarop je wordt geïncludeerd in het ziekenhuis. We loten namelijk vooraf wanneer jouw ziekenhuis zal starten met het includeren van de interventiegroep. Alle ziekenhuizen beginnen met het verzamelen van informatie over hoe de standaardzorg gaat, de controle groep. Elke 3 maanden start één ziekenhuis met de inclusie van kinderen in de interventiegroep. En

het ziekenhuis zal dit blijven doen tot het einde van de studie. Bijvoorbeeld; ziekenhuis Kikker begint in januari met de inclusie van de inventiegroep en de kinderen die mee mogen en willen doen krijgen de interventie. De andere ziekenhuizen verzamelen op hetzelfde moment nog steeds informatie over de standaardzorg. Ziekenhuis Muis start in april met het includeren van de interventiegroep, enzovoort. We weten dus van tevoren in welke groep jij zit, maar jij nog niet. Als je wilt weten waarom, kan je dit aan je ouders of de onderzoeker vragen.

Anders dan normaal

Als je in de controlegroep zit, zal er niet veel anders zijn dan normaal. Maar we willen graag wat informatie verzamelen. We zullen dit doen wanneer je het ziekenhuis verlaat en we vragen je dan om 6 maanden later voor een studiebezoek te komen. Tussendoor zullen we een vragenlijst naar je ouder(s)/verzorger(s) sturen over welke zorg je hebt gehad. Je hebt al gelezen welke metingen we zullen doen.
Als je in de interventiegroep zit, betekent dit dat er vaste tijdstippen zijn gepland waarop we informatie zullen verzamelen over wat je eet, hoe je groeit en hoe het eten gaat. Ook kijken we of je ouder(s)/verzorger(s) gestrest zijn. Dit doen we om samen met een team van verschillende experts een speciaal voedingsplan te maken voor jou.

Over de behandeling

Bezoeken

Het ligt eraan in welke groep je zit of je vaker/extra naar het ziekenhuis moet komen. Hieronder een overzicht van de studiebezoekjes.

1. Controlegroep: we zien je graag terug 6 maanden nadat je uit het ziekenhuis bent gekomen. Je ouder(s)/verzorger(s) zullen wat extra informatie meekrijgen om in te vullen voor in de tussentijd.
2. Interventiegroep: we zien jou en je ouder(s)/verzorger(s), in ieder geval voor de studie graag terug rond 6, 12 en 18 weken nadat je uit het ziekenhuis bent gekomen. En we willen je nog een keer terug zien 6 maanden na ontslag.

Metingen en vragenlijsten

Voor elk studiebezoek willen we jou en je ouder(s)/verzorger(s) vragen en aantal dingen te doen:

- Een 3-daags eetdagboek: Hierin vul jij samen met je ouder(s)/verzorger(s) in wat je 3 dagen hebt gegeten en gedronken.
- Een aantal vragenlijsten: deze vullen je ouder(s)/verzorger(s) in.

Tijdens de bezoeken willen we een aantal metingen doen:

- Gewicht, lengte en bovenarmomtrek.
- Om te meten hoeveel vet je hebt gebruiken we verschillende methodes: De huidplooi, bio-elektrische impedantie analyse (BIA) en in het Sophia kinderziekenhuis hebben we ook nog een ander apparaat waarmee we dit

kunnen meten namelijk de BODPOD . Alle drie de metingen doen geen pijn. Je kunt in onderstaande tabel lezen wat deze metingen inhouden.

Meting	Uitleg
**Huidplooi**	
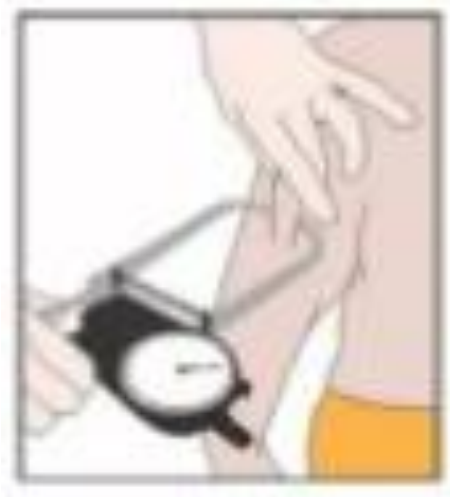	Met de huidplooimeter kan de dikte van een huidplooi worden bepaald zoals je kan zien op het plaatje. Met de verschillende huidplooimetingen kunnen we schatten hoeveel vet je hebt. Dit vergelijken we dan met getallen van kinderen met dezelfde leeftijd als jij en weten we ongeveer hoeveel vet en spiermassa je hebt.
**Bio-elektrische impedantie (BIA)**	
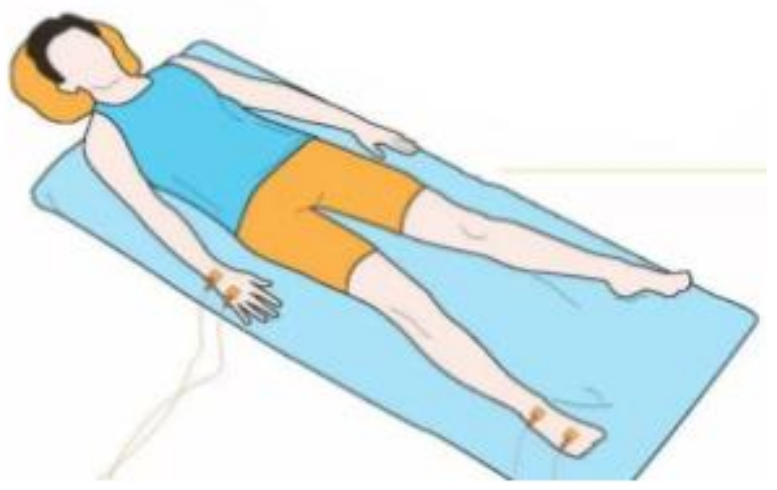	Bij de BIA wordt ook gemeten hoeveel vet je hebt. Er worden dan plakkers met elektroden op jouw hand en voet geplakt. En de gegevens die hieruit komen zetten we in een formule en hierdoor weten we ongeveer jouw vet- en spiermassa.
**BODPOD**	
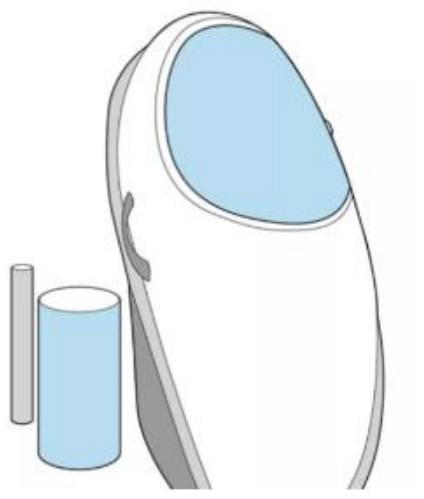	Een BODPOD is een cabine waar je in gaat zitten. De BODPOD bepaalt binnen enkele minuten het volume van jouw lichaam en kan vervolgens door middel van moeilijke formules zeggen hoeveel vet en spiermassa je ongeveer hebt.

Door deze metingen krijgen we informatie die we gelijk zullen gebruiken in de adviezen die je mee naar huis krijgt.

Ongemakken

Er zullen géén vervelende/pijnlijke dingen gebeuren voor het onderzoek, zoals bloedprikken.

Belangrijk om te weten

De informatie die we verzamelen is ‘gecodeerd’. Dat betekent dat jouw gegevens onder een nummer worden opgeslagen en dat wij niet weten dat het jouw informatie is.

Voordelen en nadelen:

- Je helpt mee aan wetenschappelijk onderzoek, en helpt daardoor de zorg telkens een beetje beter te maken.
- 6 maanden na het ontslag uit het ziekenhuis gaan we kijken hoe je groeit, maar ook hoeveel spieren je hebt.

- Een nadeel is dat je een extra bezoekje moet brengen aan het ziekenhuis 6 maanden na ontslag uit het ziekenhuis.
- Als je in de interventiegroep komt moeten je ouder(s)/verzorger(s) vragenlijsten invullen en zullen daar tijd aan kwijt zijn.

Vergoeding
Je krijgt geen vergoeding als je meedoet aan dit onderzoek. Wel krijgen je ouder(s)/verzorger(s) een vergoeding voor de (extra) reiskosten.

Informeren huisarts en behandelend arts
Wij laten je huisarts en dokters in het ziekenhuis weten dat je meedoet aan het onderzoek. Dit is voor jouw eigen veiligheid. Als je hiervoor geen toestemming geeft, kun je niet meedoen aan het onderzoek.

Einde van het onderzoek
Jouw deelname aan het onderzoekt stopt als:

- Als alle studiebezoeken geweest zijn. Het laatste bezoek is 6 maanden na je ontslag uit het ziekenhuis
- Jij zelf of je ouder(s)/verzorger(s) kiezen om te stoppen
- De onderzoeker het beter vindt om te stoppen

Het hele onderzoek is afgelopen als alle deelnemers klaar zijn. Maar na 6 maanden stopt het onderzoek voor jou en zal indien nodig de voedingsadviezen vervolgd worden bij de diëtist in het ziekenhuis waar jij de kinderarts hebt. Na het verwerken van alle gegevens zal de onderzoeker jou en je ouder(s)/verzorger(s) op de hoogte brengen van de belangrijkste uitkomsten van het onderzoek. Het kan zijn dit dat pas na een paar jaar bekend wordt. Deze informatie gaat over de hele studie en niet over jouw persoonlijke resultaten.

Jouw rechten
Moet je meedoen?
Nee, je mag **zelf weten** of je meedoet. Als je niet wilt meedoen, dan hoeft dit niet, ook als je ouder(s)/verzorger(s) dat liever wel willen. Als je wilt meedoen, zet je je handtekening op het formulier. Ook daarna **mag je altijd nog stoppen**, als je liever niet meer wilt meedoen. Vertel dit dan wel aan de onderzoeker. Je hoeft niet uit te leggen waarom je niet mee wilt doen of wanneer je besluit te stoppen.

Toestemming intrekken
Als je wilt stoppen, vertel je dit aan de onderzoeker. Dit heet: je toestemming intrekken. De informatie die al is verzameld gebruiken we nog wel voor het onderzoek.

Jouw gegevens

Voor het onderzoek hebben verschillende gegevens van je nodig:

Persoonsgegevens = informatie over wie jij bent, bijvoorbeeld je geboortedatum en waar je woont.	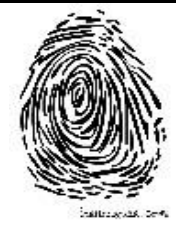
Medische gegevens = (ook een soort persoonsgegevens) informatie over je gezondheid, bijvoorbeeld of je ziek bent en of je medicijnen gebruikt.	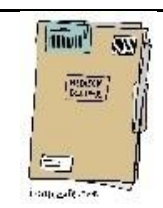
Groei gegevens en lichaamssamenstelling = We zullen je lengte en gewicht meten tijdens de bezoeken aan het ziekenhuis. Maar ook zullen wij meten met een apparaat hoe jouw lichaam is opgebouwd. Dan kijken wij hoeveel vet jij hebt.	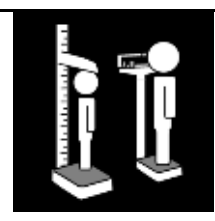
Voedingsgegevens = We zullen berekenen en verzamelen wat je aan voeding nodig hebt en wat je hebt gegeten.	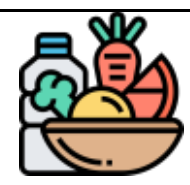
Vragenlijsten = we zullen vragen aan je ouder(s)/verzorger(s) of zij een aantal vragenlijsten willen invullen over stress en kwaliteit van leven als jij voedingszorg krijgt.	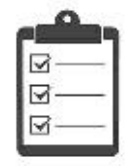

Deze **vijf dingen zijn nodig bij het doen van het onderzoek**. Je ouder(s)/verzorger(s) geven toestemming zodat we deze informatie mogen gebruiken. Wil je meer weten over wat we precies doen met jouw gegevens? Vraag het dan aan je ouder(s)/verzorger(s), het staat in hun informatiebrief. Je kunt het ook aan de onderzoeker vragen.

Verzekering

Je bent niet extra verzekerd voor dit onderzoek. Want meedoen aan het onderzoek heeft geen extra risico's. Daarom hoeft het ErasmusMC van de METC geen extra verzekering af te sluiten.

Jouw beslissing

Het formulier

Wil je meedoen? Dan zet je een handtekening op het toestemmingsformulier. We hebben ook een handtekening van jouw ouders/voogd nodig.
Ook kun je kiezen of je we je later mogen vragen voor een volgend onderzoek. We geven je later informatie over het nieuwe onderzoek, daarna hoef je pas te beslissen of je wilt meedoen.

Meer weten?

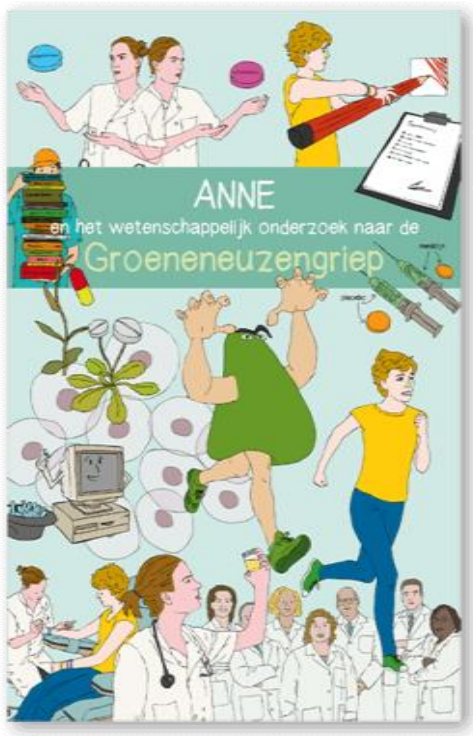

Wil je meer weten over onderzoek of over jouw rechten?

Kijk dan op www.kindenonderzoek.nl

Op deze website staat ook de strip ‘Anne en de Groeneneuzengriep’ over onderzoek.

Bijlage A: Contactgegevens

Hoofdonderzoeker:

Dr. S.C.A.T Verbruggen
Afdeling IC neonatologie & kinderen, Sophia kinderziekenhuis
Erasmus MC medisch centrum, Rotterdam
s.verbruggen@erasmusmc.nl
010-7036044

Onafhankelijk onderzoeker

Dr. B.A.E de Koning
Afdeling kindergeneeskunde, kindermaag- darm- en
leverziekten Erasmus MC medisch centrum, Rotterdam

Onderzoeker

E.J van Steenbergen
Afdeling IC neonatologie & kinderen, Sophia kinderziekenhuis
Erasmus MC medisch centrum, Rotterdam
Nutricad@erasmusmc.nl

Klachten:

Indien je niet tevreden bent over het onderzoek of de behandeling, kan je terecht bij de onafhankelijke klachtenopvang/klachtenfunctionaris van het Erasmus MC.
Op de website van het Erasmus MC is een Digitaal klachtenformulier beschikbaar via https://www.erasmusmc.nl/nl-nl/patientenzorg/klachtenopvang-en-klachtenbemiddeling
Na het invullen wordt het formulier automatisch verzonden naar de klachtenfunctionaris.
Als het niet lukt om het digitale klachtenformulier in te vullen, dan kan je je klacht ook per post versturen: Erasmus MC, Secretariaat Klachtenopvang (GK-745), Antwoordnummer 55, 3000 WB Rotterdam.
Vermeld in de brief je naam, patiëntnummer (indien van toepassing), naam van het onderzoek en contactgegevens. Na ontvangst van de brief zal de klachtenfunctionaris contact met je opnemen.

Functionaris voor de Gegevensbescherming van de instelling:

De Functionaris voor de Gegevensbescherming van het Erasmus MC is bereikbaar via het secretariaat van de afdeling Juridische Zaken. E-mail: Functionaris.gegevensbescherming@erasmusmc.nl, Tel: 010-703 4986

Voor meer informatie over jouw rechten:
Voor meer informatie of bij vragen over jouw rechten kun je contact opnemen met de functionaris voor de Gegevensbescherming of met de Autoriteit Persoonsgegevens.

Toestemmingsformulier voor kinderen van 12-16 jaar

Onderzoek naar voedingszorg na ontslag uit het ziekenhuis (NutriCAD) bij kinderen

- Ik heb de informatie **begrepen**. Ook kon ik vragen stellen. Mijn **vragen zijn beantwoord**.
- Ik had **genoeg tijd om te beslissen** of ik meedoe.
- Ik weet dat ik **niet verplicht** ben om mee te doen.
- Ik begrijp dat ik **altijd mag stoppen** als ik niet meer mee wil doen.
- Ik geef toestemming **voor het informeren** van mijn huisarts en specialist(en) die mij behandelt dat ik meedoe aan dit onderzoek.

Kruis jouw antwoord aan

Ik geef toestemming voor het gebruik van gegevens uit mijn patiëntendossier voor dit onderzoek<br>*(Als je hier kiest voor 'nee', dan kun je niet meedoen aan dit onderzoek)*	☐ Ja<br>☐ Nee
Ik geef toestemming om mij later te vragen voor vervolgonderzoek	☐ Ja<br>☐ Nee

Ik wil meedoen aan dit onderzoek.

Naam deelnemer:

Handtekening: Datum : __ / __ / __

<u>Dit stuk is voor de onderzoeker:</u>

Ik verklaar dat ik deze proefpersoon volledig heb geïnformeerd over het genoemde onderzoek. Als er tijdens het onderzoek informatie bekend wordt die de toestemming van de proefpersoon zou kunnen beïnvloeden, dan breng ik hem/haar daarvan tijdig op de hoogte.

Naam onderzoeker (of diens vertegenwoordiger):

Handtekening: Datum: __ / __ / __

<*indien van toepassing*> Aanvullende informatie is gegeven door:
Naam:
Functie:

Handtekening: Datum: __ / __ / __

De proefpersoon krijgt een volledige informatiebrief mee, samen met een getekende versie van het toestemmingsformulier.
