# Supplementary material for: Nutritional Care After Hospital Discharge (NutriCAD): protocol for a stepped-wedge, multicentre, randomised controlled trial in paediatrics
Source: BMJ Open. 2026 Oct 27;16(10):e122183. doi: 10.1136/bmjopen-2026-122183 (PMC13723836; doi:10.1136/bmjopen-2026-122183)
Supplement: Supplementary file 3 [file bmjopen-2026-122183.reviewer_comments.pdf]

PEER REVIEW HISTORY

BMJ Open publishes all reviews undertaken for accepted manuscripts. Reviewers are asked to complete a checklist review form and are provided with free text boxes to elaborate on their assessment. These free text comments are reproduced below.

ARTICLE DETAILS

Title (Provisional)

Nutritional Care After Hospital Discharge (NutriCAD): protocol for a stepped-wedge, multicentre, randomised controlled trial in paediatrics

Authors

van Steenbergen, Esther; Hage, Doortje; Lebon, Ankie; van Wering, Herbert; Stapelbroek, Janneke; Winder-Bakker, Eefje; Bottema, Renske; Baart-Visser, Merel; Van Gils, Sandra; Van den Berg, Evi; Kroon, Esmée; Soldaat, Yolah; Poley, Marten J.; Willemsen, Sten; Hulst, Jessie; Kindermann, Angelika; Dulfer, K.; van der Louw, Elisabeth Johanna Theresia Maria; Joosten, K; Verbruggen, Sascha

VERSION 1 - REVIEW

**Reviewer**	**1**
**Name**	**Choi, Seong-Woo**
**Affiliation**	**Chosun University College of Medicine, Prevent Medicine**
**Date**	**02-Jul-2026**
**COI**	**None**

This manuscript presents a protocol for a randomized controlled trial evaluating the effectiveness of a nutritional management intervention for children after hospital discharge. The study addresses an important clinical issue, and the proposed methodology appears promising. However, several issues should be considered to improve the clarity and rigor of the study protocol.

Study design

• The study involves one academic hospital and five general hospitals. Please provide information comparing the participating hospitals in terms of hospital size, number of pediatric patients, level of care, and other relevant characteristics to demonstrate their comparability.

• As shown in Figure 1, the recruitment periods for the intervention and control groups differ across hospitals. For example, Hospital 1 has a relatively short recruitment period for the control group and a much longer period for the intervention group, whereas Hospital 6

shows the opposite pattern. To minimize potential bias arising from this design, it is important to demonstrate that the participating hospitals are comparable with respect to patient volume, patient characteristics, and standards of care. Given that the study includes both an academic hospital and general hospitals, additional explanation regarding inter-hospital comparability and strategies to address potential center effects would be helpful.

Intervention

• Please provide a more detailed description of the parent empowerment program. It is unclear whether the intervention consists solely of providing educational materials or includes direct parent education sessions. If educational sessions are conducted, please describe the content, duration, frequency, mode of delivery, and qualifications of the instructors or healthcare professionals involved.

Usual care

• Rather than simply stating that usual care differs across hospitals, it would be helpful to describe the type of care typically provided at each site, particularly with regard to nutritional management and parental education after discharge. This would allow readers to better understand the differences between the intervention and usual care groups.

Terminology and outcome measures

• The terminology used in Figure 2 and Table 2 appears to be inconsistent, which may cause confusion. Please consider using consistent terminology throughout the manuscript.

• In addition, descriptions of the study outcomes and questionnaires should be included in the main text rather than being presented only in the Supplementary Materials. Clear descriptions of each instrument, including its purpose, measurement method, and interpretation, would improve the readability of the manuscript.

**Reviewer**	**2**
**Name**	**Cheong, Jeanie**
**Affiliation**	**Royal Women's Hospital, Newborn Research**
**Date**	**07-Jul-2026**
**COI**	**None**

This is a protocol for a nutritional support intervention, conducted post hospital discharge across 6 hospitals in the Netherlands. A stepped-wedge cluster randomised controlled design is used, with the anticipated completion to be at the end of 2026. A protocol amendment was introduced 9 months into recruitment due to slower-than anticipated recruitment rates. The authors presented a revised increase in the power of the study as a result of this amendment from 39% to 53%.

This study addresses an important topic, and is clearly presented. The design is appropriate for the research question. I have a few comments for the authors to consider:

1) A big drawback for this study is the lack of lived experience consumer involvement in the design of the study. This is especially pertinent given that a component of the intervention involves a parent empowerment program. Another lost opportunity to understand the consumer experience is in the nested substudy to understand barriers and facilitators for future implementation. Understanding this from the perspectives of the parents and participants themselves is an important aspect of successful implementation esp. acceptability and compliance. These points need to be acknowledged as limitations in the discussion.

2) Parent empowerment program. I note that the recruitment age range is wide i.e. up to 18 years. Some participants will be in their mid-late adolescence, and some within the age range of giving their own consents. Sections that specifically refer to parents need to also have specific details as how these procedures apply to the older participants that are eligible for recruitment. E.g. is there a "participant empowerment program" if the trial participant is 18 years' of age?

3) Statistical analyses. The authors refer to adjustment of models for a list of predefined confounders. With this in mind, it would be useful to present a Directed Acyclic Graph (DAG) to provide clarity as to how these confounders have been determined. I do appreciate that DAGs are not strictly required for randomised trials, but in this case, a DAG would be informative.

4) Power of the study. This is an important limitation to reaching a meaningful conclusion, which the authors have acknowledged to a degree in the discussion. Can the authors provide more explanation as to why the trial was originally designed with an even lower power in mind?

VERSION 1 - AUTHOR RESPONSE

Reviewer 1

The study involves one academic hospital and five general hospitals. Please provide information comparing the participating hospitals in terms of hospital size, number of pediatric patients, level of care, and other relevant characteristics to demonstrate their comparability.

Response: We thank the reviewer for this helpful suggestion. We have added a comparison of participating hospitals with respect to annual paediatric admissions, level of care, and site characteristics in Supplementary Table 1, and we now refer to this table in the Study design section. We note that not all participating sites were able to provide exact admission figures, as centralised paediatric admissions data are not uniformly recorded or readily accessible across Dutch general hospitals. Where precise numbers were unavailable, site-level qualitative information on level of care, dietitian availability, and usual discharge practice has been included to allow a meaningful comparison. Despite this limitation, the available data confirm that all five general hospitals deliver secondary-level paediatric care, have dedicated paediatric dietitians available on weekdays, and

share a comparable patient pathway for nutritionally at-risk children, supporting the feasibility of the stepped-wedge design across sites.

As shown in Figure 1, the recruitment periods for the intervention and control groups differ across hospitals. For example, Hospital 1 has a relatively short recruitment period for the control group and a much longer period for the intervention group, whereas Hospital 6 shows the opposite pattern. To minimize potential bias arising from this design, it is important to demonstrate that the participating hospitals are comparable with respect to patient volume, patient characteristics, and standards of care. Given that the study includes both an academic hospital and general hospitals, additional explanation regarding inter-hospital comparability and strategies to address potential center effects would be helpful.

Response: We agree and have revised the Study design and Statistical analysis sections. We now explain that randomisation is performed at cluster level and stratified by level of care, and that the academic hospital was placed centrally in the sequence because of its larger patient volume. We also clarified that intervention status and calendar period will be included as fixed effects and hospital as a random effect, thereby accounting for within-cluster correlation and potential centre effects. In addition, the new Supplementary Table 1 provides the requested site comparison.

Please provide a more detailed description of the parent empowerment program. It is unclear whether the intervention consists solely of providing educational materials or includes direct parent education sessions. If educational sessions are conducted, please describe the content, duration, frequency, mode of delivery, and qualifications of the instructors or healthcare professionals involved.

Response: We have expanded the Intervention section to clarify that the parent empowerment programme is not limited to written material. It consists of structured education delivered by the paediatric dietitian at discharge and at follow-up visits at 6, 12, and 18 weeks. We describe the use of accessible written and digital support, the focus on the child's nutritional needs and feeding plan, and adaptation to participant self-management for participants aged 16 years and older.

Rather than simply stating that usual care differs across hospitals, it would be helpful to describe the type of care typically provided at each site, particularly with regard to nutritional management and parental education after discharge. This would allow readers to better understand the differences between the intervention and usual care groups.

Response: We have revised the Usual Care section. We now describe that, in general, usual care consists of a written discharge letter with nutritional instructions to the general practitioner and/or community dietitian, without a structured multidisciplinary outpatient follow-up programme. We also clarify that at the academic centre community dietitians receive a more detailed transfer document, whereas in the general hospitals discharge instructions vary by ward and clinician. Information per hospital is provided in Supplementary Table 1.

The terminology used in Figure 2 and Table 2 appears to be inconsistent, which may cause confusion. Please consider using consistent terminology throughout the manuscript.

Response: We have reviewed and harmonised terminology across the manuscript, Figure 2, and Table 2. In particular, terminology for nutritional intake/support, feeding behaviour, parental psychological outcomes, health-related quality of life, healthcare utilisation, and productivity losses has been made consistent where these outcomes are described.

In addition, descriptions of the study outcomes and questionnaires should be included in the main text rather than being presented only in the Supplementary Materials. Clear descriptions of each instrument, including its purpose, measurement method, and interpretation, would improve the readability of the manuscript.

Response: We have added a summary table in the main text listing the study outcomes, instruments, respondents, and assessment time points. We also added explanatory text to improve readability and retained more detailed measurement protocols and questionnaire descriptions in the Supplementary Materials.

Reviewer 2

A big drawback for this study is the lack of lived experience consumer involvement in the design of the study. This is especially pertinent given that a component of the intervention involves a parent empowerment program. Another lost opportunity to understand the consumer experience is in the nested substudy to understand barriers and facilitators for future implementation. Understanding this from the perspectives of the parents and participants themselves is an important aspect of successful implementation esp. acceptability and compliance. These points need to be acknowledged as limitations in the discussion.

Response: We agree with the reviewer and have added this explicitly to the Discussion as a limitation. We now state that the study did not include formal patient and public involvement in its design, and that the absence of lived-experience input from patients and families is a recognised limitation, particularly given the parent empowerment component and the nested qualitative implementation substudy. We also added that future confirmatory work should incorporate structured patient and public involvement from the outset.

Parent empowerment program. I note that the recruitment age range is wide i.e. up to 18 years. Some participants will be in their mid-late adolescence, and some within the age range of giving their own consents. Sections that specifically refer to parents need to also have specific details as how these procedures apply to the older participants that are eligible for recruitment. E.g. is there a 'participant empowerment program' if the trial participant is 18 years of age?

Response: Thank you for this important point. We have revised the manuscript to specify how consent and empowerment procedures apply to older participants. For participants aged 16 years and older, informed consent is obtained from the participant themselves, and the empowerment component is adapted to focus on participant nutritional self-management rather than only parent-directed education.

Statistical analyses. The authors refer to adjustment of models for a list of predefined confounders. With this in mind, it would be useful to present a Directed Acyclic Graph (DAG) to provide clarity as to how these confounders have been determined. I do appreciate that DAGs are not strictly required for randomised trials, but in this case, a DAG would be informative.

Response: We thank the reviewer for this constructive suggestion. We have clarified in the Statistical analysis section which predefined confounders will be considered in adjusted models, including age, illness type and severity, nutritional status at admission, and relevant nutritional risk scores. We have added a Directed Acyclic Graph (Figure 3) to clarify the rationale for covariate adjustment. Confounding of the treatment–outcome relationship by patient-level characteristics is not expected given randomization. However, because this is a stepped-wedge cluster-randomized design, calendar time and cluster (hospital) act as genuine confounders: exposure status is mechanically linked to time-since-trial-start and to cluster-specific crossover timing, and both may independently influence outcomes through secular trends and case-mix or practice variation, respectively. Our models therefore adjust for time period and cluster to address this design-induced confounding. The four patient-level variables listed in our original description (age, illness type and severity, nutritional status at admission, and nutritional risk score) are included not as confounders but as prognostic covariates, to improve precision of the treatment effect estimate, consistent with recommended practice for covariate adjustment in randomized trials [FDA 2023; Tsiatis et al. 2008]. The DAG makes this distinction explicit.

Power of the study. This is an important limitation to reaching a meaningful conclusion, which the authors have acknowledged to a degree in the discussion. Can the authors provide more explanation as to why the trial was originally designed with an even lower power in mind?

Response: We have expanded the Sample size section and Discussion to explain this more transparently. The original stepped-wedge schedule was designed around the maximum feasible sample size within the available funding period and projected patient volume across participating hospitals. The low effective power was therefore a consequence of pragmatic and financial

constraints and slower-than-anticipated recruitment, not a predefined target. The protocol amendment adding two mid-study inclusion periods increased the estimated effective power from 39% to 53%. We now state explicitly that the trial should be interpreted as a necessary first empirical step in an understudied area, intended to inform a future, adequately powered confirmatory trial.

VERSION 2 - REVIEW

**Reviewer**	**1**
**Name**	**Choi, Seong-Woo**
**Affiliation**	**Chosun University College of Medicine, Prevent Medicine**
**Date**	**01-Oct-2026**
**COI**	

none
