# Supplementary material for: Nutritional Care After Hospital Discharge (NutriCAD): protocol for a stepped-wedge, multicentre, randomised controlled trial in paediatrics
Source: BMJ Open. 2026 Oct 27;16(10):e122183. doi: 10.1136/bmjopen-2026-122183 (PMC13723836; doi:10.1136/bmjopen-2026-122183)
Supplement: Supplementary file 4 [file bmjopen-16-10-s001.docx]

**Supplementary Material A**

**Detailed description of secondary outcome measures**

***Body composition***

Body composition will be assessed at each outpatient visit using a standardised multi‑method approach. Triceps skinfold thickness will be measured in triplicate with a calibrated Harpenden calliper following ISAK guidelines [28,29]. MUAC will be used to calculate upper arm muscle and fat area. Where available, InBody bioelectrical impedance analysis will provide segmental fat mass (FM) and fat‑free mass (FFM) estimates [30]. At Erasmus MC – Sophia only, whole‑body FM and FFM will also be measured using air displacement plethysmography (Pea Pod® for infants; Bod Pod® for children ≥2 years) according to validated protocols [31,32].

***Duration and characteristics of nutritional support***

Type, duration, and frequency of all nutritional support (oral supplements and enteral tube feeding), including NGT dependency and formula type will be prospectively documented by the dietitian. Time to complete discontinuation of nutritional support will serve as a time‑to‑event secondary endpoint.

***Nutritional requirements and dietary intake***

Energy and protein requirements will be estimated using the Dutch guideline *Screening and Treatment of Malnutrition in Children* (2022), which incorporates STRONGkids, MUST, and NRS‑2002 tools [33]. Other nutrient recommendations follow Dutch Health Council reference values. Dietary intake will be evaluated using a three‑day food diary completed before each follow‑up visit. A registered dietitian will analyse diaries using Evry© (2023) to derive energy intake, macronutrients (protein g/kg and %E; fat %E; carbohydrate %E), and key micronutrients.

***Child feeding behaviour***

Eating behaviour will be assessed using validated parent‑completed questionnaires: the BEBQ for infants <12 months [34] and the CEBQ for children ≥12 months [35].

***Parental psychological outcomes***

Distress will be measured with the Distress Thermometer for Parents (DT‑P), a validated single‑item scale [36]. PTSD symptoms will be screened using the PCL‑5, a 20‑item scale with a cut‑off ≥31 for probable PTSD, yielding total and four domain scores (intrusion, avoidance, negative mood/cognition, hyperarousal) with a 30‑day recall period [37]. Anxiety and depression will be assessed using PROMIS Short Forms (Anxiety 8a; Depression 8b), each with eight items and a seven‑day recall period. Raw scores are converted to T‑scores (mean 50, SD 10), with higher scores indicating greater symptom burden [38].

***Child health‑related quality of life and functioning***

HRQoL and functioning across physical, emotional, social, and cognitive domains will be measured using the GPROM instrument suite recommended in the Dutch national advisory report [39]. The CHU9D (validated for ages 5–17) will also be used to generate utility scores for QALY calculations [43–45].

***Economic evaluation***

A within‑trial cost‑effectiveness analysis (CEA) from a societal perspective and a budget impact analysis (BIA) from the healthcare payer perspective will be conducted following ISPOR guidelines [40]. The CEA will estimate incremental cost per QALY gained and per unit of growth improvement. Cost categories include direct medical costs (readmissions, outpatient visits, home enteral nutrition) and indirect costs (parental productivity losses, behavioural management, school absenteeism). Healthcare utilisation will be measured using the iMCQ [41], and productivity losses using the iPCQ [42]. HRQoL data from GPROM and CHU9D will inform QALY calculations.

**Harms**

The NutriCAD intervention consists of nutritional counselling and structured multidisciplinary follow-up; no investigational medicinal product is administered. The risk of study-related harm is therefore considered negligible, and formal AE/SAE reporting to the METC has been waived by the accredited ethics committee (Erasmus MC, MEC-2023-0490). In accordance with the Dutch WMO, the study will be temporarily halted if the coordinating investigator judges that the disadvantages of participation are significantly greater than foreseen. Should a participant require hospitalisation during the follow-up period, this will be recorded as a clinical event but will not be attributed to the study intervention unless a direct causal relationship is established by the investigator. The absence of a formal DSMB is justified by the non-invasive nature of the intervention and the negligible safety risk profile. No interim efficacy analysis is planned.

**Data management**

All data are collected and managed within the secure ICT infrastructure of Erasmus MC. Questionnaire data are captured electronically and stored automatically in a validated digital database; anthropometric and body composition measurements are entered by trained site personnel using site-specific Standard Operating Procedures. All personal data are pseudonymised immediately upon entry: participants are assigned a unique study code, and the linkage key is held exclusively by the principal investigator at the coordinating site. Data from participating hospitals are transferred to the coordinating centre via secure, encrypted channels in accordance with the Erasmus MC data transfer agreements. Range checks and logical validation rules are applied at data entry to detect implausible values. Data will be stored securely for a minimum of 15 years after study completion, in compliance with the EU General Data Protection Regulation (GDPR) and the Dutch Act on Implementation of the GDPR (UAVG). Access to identifiable data is restricted to the direct research team; monitors from the Research Office Sophia conduct periodic monitoring visits in accordance with the NFU risk-classification (negligible risk) monitoring framework and Erasmus MC standard operating procedures.

**Trial monitoring**

Monitoring is conducted by independent monitors from the Research Office Sophia, Erasmus MC, in accordance with applicable Dutch law and regulation, the NFU Guideline Quality Assurance of Research Involving Human Subjects, and Erasmus MC standard operating procedures. The trial is classified as negligible risk under the NFU risk-classification framework. A study-specific monitoring plan was approved by the coordinating investigator prior to first enrolment. An initiation meeting was held with all participating sites before the first inclusion. Monitors have no involvement in the design, conduct, or interpretation of the study. An independent Data and Safety Monitoring Board (DSMB) was not established, as the intervention is non-pharmacological and non-invasive and the ethics committee has waived SAE reporting requirements. Annual progress reports are submitted to the accredited METC (Erasmus MC). All protocol amendments are communicated to the METC and, where applicable, to the trial registry and participating sites prior to implementation.
