# Supplementary material for: Nutritional Care After Hospital Discharge (NutriCAD): protocol for a stepped-wedge, multicentre, randomised controlled trial in paediatrics
Source: BMJ Open. 2026 Oct 27;16(10):e122183. doi: 10.1136/bmjopen-2026-122183 (PMC13723836; doi:10.1136/bmjopen-2026-122183)
Supplement: Supplementary file 5 [file bmjopen-16-10-s004.docx]

**Supplementary Table 1. Hospital characteristics and usual care**

| **Hospital** | **Erasmus MC – Sophia Children’s Hospital** | **Albert Schweitzer Hospital** | **Amphia** | **Catharina ziekenhuis (CZE)** | **Noordwest Hospital Group** | **Hospital Gelderse** |
| --- | --- | --- | --- | --- | --- | --- |
| **City** | Rotterdam | Dordrecht | Breda | Eindhoven | Alkmaar | Ede |
| **Approx. annual paediatric admissions 2025** | 5700 | 2300 | 2800 | 1239 | 1660 | 1500 |
| **Hospital type** | Academic | General hospital | General hospital | General hospital | General hospital | General hospital |
| **Level of paediatric care** | Tertiary care | Secondary care, top clinical | Secondary care, top clinical | Secondary care, top clinical | Secondary care, top clinical | Secondary care |
| **PICU / tertiary services** | Yes | No | No | No | No | No |
| **Paediatric dietitian availability** | Weekdays/ dedicated paediatric dietitian | Weekdays/dedicated paediatric dietitian | Weekdays/dedicated paediatric dietitian | Weekdays/dedicated paediatric dietitian | Weekdays/dedicated paediatric dietitian | Weekdays/dedicated paediatric dietitian |
| **Usual discharge nutritional care** | Depending on diagnosis, if tertiary diagnosis follow-up remains in EMC. If not follow-up in secondary or primary care. Frequency differce per patient. | Patients who receive dietetic care during hospitalization are frequently scheduled for outpatient follow-up after discharge.  No standardized guidelines exist regarding the timing of the initial dietetic consultation during admission.  Subsequent nutritional care may be provided in primary, secondary, or tertiary care settings, depending on the patient's clinical needs and referral criteria. | Medical feeding (recommended by dietitian), tube feeding. Appointments with the paediatrician and dietitian after thischarge. Depending on child, growth and how parents feel | Following hospital discharge, nutritional follow-up may be provided by a dietitian in either primary or secondary care, depending on the applicable outpatient referral criteria. | We follow up with patients from the pediatric ward ourselves after discharge. We only refer children who are overweight or have certain simple intolerances to primary care. | Following hospital discharge, nutritional follow-up may be provided by a dietitian in either primary or secondary care, depending on the applicable outpatient referral criteria. |
| **Parent / participant education in usual care** | Depending on diagnosis and diet. Providing educational materials if necessary and/or creating a personalized dietary plan. Arrange medical nutrition for home use. | Providing educational materials if necessary and/or creating a personalized dietary plan. Arrange medical nutrition for home use. | Providing educational materials if necessary and/or creating a personalized dietary plan. Teaching tube feeding before discharge. Arrange medical nutrition for home use. | Written dietary information is provided at discharge when nutrition is relevant to the underlying condition.  Educational materials may include guidance on nutritional management in inflammatory bowel disease (IBD) or recommendations for energy-enriched diets.  Since the introduction of the NutriCAD study, information leaflets on energy- and protein-enriched nutrition have been distributed routinely. | Providing educational materials if necessary and/or creating a personalized dietary plan. Arrange medical nutrition for home use. | Providing educational materials if necessary and/or creating a personalized dietary plan. Arrange medical nutrition for home use. |
| **Structured multidisciplinary follow-up before NutriCAD?** | Partial, depending on diagnosis. | Partial, depending on diagnosis | We collaborate with other disciplines and consult with them regularly. No structured multidisciplinary consultation has been organized following discharge. | Partial, depending on diagnosis | We collaborate with other disciplines and consult with them regularly. No structured multidisciplinary consultation has been organized following discharge. | Partial, depending on diagnosis |
| **Existing transfer to GP / community dietitian** | Via discharge letter and if neccesarily by phone | Referrals to primary care dietitians were already common practice, and collaboration with these professionals was generally well established. | Referrals to primary care dietitians were already common practice, and collaboration with these professionals was generally well established. | Referrals to primary care dietitians were already common practice, and collaboration with these professionals was generally well established. | No, we don't do this often, because we don't often refer patients to primary care. | Referrals to primary care dietitians were already common practice, and collaboration with these professionals was generally well established. |
